# Phylogenetic Analysis and Genetic Diversity of *Colletotrichum falcatum* Isolates Causing Sugarcane Red Rot Disease in Bangladesh

**DOI:** 10.3390/biology10090862

**Published:** 2021-09-03

**Authors:** Md Imam Hossain, Khairulmazmi Ahmad, Ganesan Vadamalai, Yasmeen Siddiqui, Norsazilawati Saad, Osumanu Haruna Ahmed, Erneeza Mohd Hata, Fariz Adzmi, Osamah Rashed, Muhammad Ziaur Rahman, Abdulaziz Bashir Kutawa

**Affiliations:** 1 Department of Plant Protection, Faculty of Agriculture, Universiti Putra Malaysia, Serdang 43400, Selangor, Malaysia; imam4all@gmail.com (M.I.H.); ganesanv@upm.edu.my (G.V.); norsazilawati@upm.edu.my (N.S.); erneeza@upm.edu.my (E.M.H.); osamahrashid@gmail.com (O.R.); ziapath@gmail.com (M.Z.R.); abashir@fudutsinma.edu.ng (A.B.K.); 2 Pathology Division, Bangladesh Sugarcrop Research Institute (BSRI), Ishurdi 6620, Pabna, Bangladesh; 3 Laboratory of Sustainable Agronomy and Crop Protection, Institute of Plantation Studies, Universiti Putra Malaysia, Serdang 43400, Selangor, Malaysia; farizadzmi@upm.edu.my; 4 Department of Crop Science, Faculty of Agriculture and Food Sciences, Bintulu Campus Sarawak, Universiti Putra Malaysia, Bintulu 97008, Sarawak, Malaysia; osumanu@upm.edu.my; 5 Institut Ekosains Borneo, Bintulu Campus Sarawak, Universiti Putra Malaysia, Bintulu 97008, Sarawak, Malaysia; 6 Department of Biological Sciences, Faculty of Life Science, Federal University Dutsin-Ma, P.M.B 5001 Dutsin-Ma, Nigeria

**Keywords:** *Colletotrichum falcatum*, genetic diversity, phylogeny, red rot, *Saccharum officinarum*

## Abstract

**Simple Summary:**

Sugarcane is an important agro-industrial crop because it is one of the major sources of white sugar. Red rot which is caused by *Colletotrichum falcatum* is the most devastating disease of sugarcane because its infestation results in significant sugarcane yield loss. The intra- and inter-specific genetic diversity, population structure and phylogenetic relationship amongst *C. falcatum* isolates from Bangladesh remain unclear. This information is essential for the effective management of red rot and to also develop resistant sugarcane varieties through plant breeding programmes. This paper demonstrates the phylogenetic relationship and genetic diversity of *C. falcatum* isolates from Bangladesh. Also, it provides baseline information that can be used to establish red rot disease management strategies for future application.

**Abstract:**

*Colletotrichum falcatum* Went causes red rot disease in sugarcane farming in the tropical and sub-tropical regions. This disease causes significant economic loss to the sugarcane production industry. Successful disease management strategies depend on understanding the evolutionary relationship between pathogens, genetic diversity, and population structure, particularly at the intra-specific level. Forty-one isolates of *C. falcatum* were collected from different sugarcane farms across Bangladesh for molecular identification, phylogeny and genetic diversity study. The four genes namely, ITS-rDNA, *β-tubulin*, Actin and GAPDH sequences were conducted. All the 41 *C. falcatum* isolates showed a 99–100% similarity index to the conserved gene sequences in the GenBank database. The phylogram of the four genes revealed that *C. falcatum* isolates of Bangladesh clustered in the same clade and no distinct geographical structuring were evident within the clade. The four gene sequences revealed that *C. falcatum* isolates from Bangladesh differed from other countries´ isolates because of nucleotides substitution at different loci. The genetic structure of *C. falcatum* isolates were determined using ISSR marker generated 404 polymorphic loci from 10 selected markers. The percentage of polymorphic loci was 99.01. The genetic variability at species level was slightly higher than at population level. Total mean gene diversity at the species level was 0.1732 whereas at population level it was 0.1521. The cluster analysis divided 41 isolates into four main genetic groups and the principal component analysis was consistent with cluster analysis. To the best of our knowledge, this is the first finding on characterizing *C. falcatum* isolates infesting sugarcane in Bangladesh. The results of this present study provide important baseline information vis a vis *C. falcatum* phylogeny analysis and genetic diversity study.

## 1. Introduction

Sugarcane (*Saccharum officinarum* L.) is widely cultivated in the tropics and sub-tropics. It is one of the main sources of sugar in the world [1]. Sugarcane covers approximately 26.3 million hectares of the global arable land. The total production of approximately 1.9 billion tons [2]. In Bangladesh (North West and South East of Bangladesh), sugarcane is cultivated on 0.11 million hectares of land for white sugar, ethanol, juice production, chewing, and brown sugar [3]. Sugarcane plants are susceptible to several diseases, of which red rot which is caused by *Colletotrichum falcatum* is one of the devastating diseases (Glomerallacae of Ascomycota). *Glomeralla tucumanesis* which is the sexual stage of *C. falcatum* [4], is also called as *Physalospora tucumanesis* [5]. Sugarcane red rot is endemic in the tropics and subtropics and it poses a serious challenge to sugarcane production in Bangladesh [6]. Depending on cultivars, environment, and pathogen strain, it reduces sugarcane weight up to 29% with sugar recovery loss up to 31% [7].

Although several studies had been carried out on the biochemical, physiological, molecular analysis of pathogen-plant interactions and the complete genetic basis for the progression of the disease is yet to be identified. To establish novel techniques for the successful control of the red rot disease, a clearer understanding of the phylogenetic relationship and genetic diversity are essential. Currently, the viable way of managing the disease is using resistant cultivars [8]. However, the rapidly evolving *C. falcatum* fungus contributes to the formation of new virulent strains, complicating the red rot resistance development [9]. The red rot epiphytotic had negatively impacted the widely known cultivars [10]. Frequent shifts in the pathogen’s genetic structure and incremental shortening of the genetic link of resistant origins are the main factors of the natural choice for new, potential isolates and deteriorate the resistance in the host [9].Appropriate detection and classification of isolates in *C. falcatum* tainting are crucial for the exact taxonomic identification required for disease management and breeding for resistance [11].

Detection and characterization of *C. falcatum* are carried out based on morphological characteristics such as conidia shape and size, setae or teleomorphic appearance, and characteristics of culture such as colour, rate of growth and texture [12]. However, in different environment *Colletotrichum* species can demonstrate morphological and physiological differences. Therefore, accurate identification of *Colletotrichum* species is difficult [13]. On the other hand, molecular marker techniques have improved identification precision, speed, and classification of phytopathogenic fungi [14]. Nucleotide sequences differ from species to species because of this, rDNA-Internal transcribed spacer (rDNA-ITS) region is commonly used for *Colletotrichum* spp. differentiation for various plants [15]. Phylogenetic analysis of the DNA sequence data from the gene regions of ITS-rDNA, Beta-tubulin (*β-tubulin*), Actin (ACT) and glyceraldehyde-3-phosphate dehydrogenase (GAPDH) have been used to construct species-specific primers for *C. acutatum* and *C. gloeosporioides* species complex detection and phylogenetic analysis [16]. Knowledge on the variability of pathotypes is significant in selecting the correct isolates for resistance testing in plant breeding programmes. Over the past few decades, molecular markers have been used to determine genetic variation, genetic architecture, and virulence in plant pathogen populations. Because the genetic makeup of *C. falcatum* isolates is not known, knowledge on the genetic variability of the population is important for understanding the evolutionary process and prospects to evolve for environmental change [17]. In Asia, the characterizations of *C. falcatum* for sugarcane was carried out in India using sequence analysis of ITS region and genetic diversity by inter-simple sequence repeat (ISSR), Kumar et al. [9]. Currently, there is dearth of information on red rot disease for sugarcane plantations in Bangladesh. This study aimed to isolate *C. falcatum* from infected sugarcane plantations in Bangladesh. Also, the phylogenetic characteristics and the genetic diversity among the populations of *C. falcatum* isolates were determined. The results from this study could be a platform for generating detailed information on the genetic variation of sugarcane red rot disease in Bangladesh using molecular approaches. Besides, this research provides valuable information on how to overcome the disease. Thus, the overall findings of this present study could contribute to improving sugarcane production in Bangladesh.

## 2. Materials and Methods

### 2.1. Sampling, Isolation and Pure Culture Maintenance

**Figure 1:**
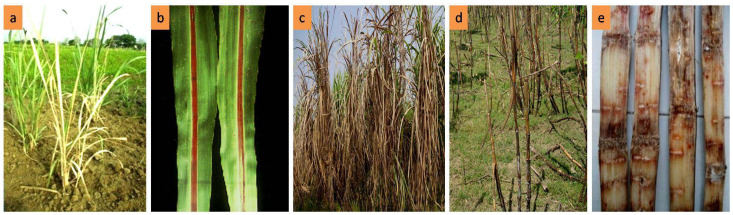
Typical Red rot disease symptoms (**a**) Tiller red rot (**b**) Lamina red rot (**c**) Red rot infected field (**d**) Stem red rot (**e**) Stem red rot internal symptom.

A survey was conducted to collect red rot infected disease samples (Figure 1) from different sugarcane growing areas in four regions (Supplementary Figure S1) in Bangladesh. The stalk of seven to ten month-old sugarcane plants with disease symptoms were collected randomly from sugarcane fields. To inspect the typical red rot symptoms, infected canes were cut open with a disinfected dagger. Afterwards, longitudinally reddish colour tissue and transverse white patches were observed (Figure 1e). The red rot pathogen was isolated as described by Rang Swami [18], and Pandey [19]. The symptomatic sugarcane tissues were cut (5 × 5 mm pieces) and surface sterilized using 1% NaOCl for 1 min followed by dipping into 70% ethanol for 1 min. The sterilized tissues were rinsed three times with sterilized water and dried on sterilized Whatman filter paper [20]. Thereafter, the samples were placed on Petri plates containing potato dextrose agar (PDA, Merck, Germany). All the plates were incubated at 28–30 °C for fungal growth. The grown margins of the fungal hyphae produced by the tissues were transferred to the fresh PDA after five days of incubation. The spores’ mass was picked using a sterilized wire loop and streaked on to the water agar. The hyphal tips of single germinated spores were transferred to the PDA slants to keep the pure colonies viable. Pure cultures were maintained on PDA plates with periodic sub-culture and kept at 4 ± 1 °C. Forty-one *C. falcatum* isolates were obtained from 14 sub-tropical sugarcane cultivars (Table 1).

### 2.2. Morphological and Colonies Variability

Colony characteristics were examined after which data were collected from the cultures grown on PDA [19]. Each of the isolates was cultured on PDA plates and incubated at 28 ± 2 °C. Diameter of the colony was measured daily for seven days after which the growth rate was measured as the average daily growth rate of the seven days. Colony characters, colour, topography, and margin shape were recorded on the 10th day of the incubation [21,22]. For each isolate, three replicates of the culture were evaluated. The spores of each isolate were collected in a sterilized conical flask with distilled water (100 mL) to determine strength of the sporulation. The suspension was blended for three minutes in a warring blender, after which the concentration of the purified conidia (100 mL) was measured using a haemocytometer [23].

Three replicates were used for each isolate. The shape of the conidia was observed under a high power compound microscope during which the conidia length and width were measured using Dino Capture 2.0 software, after connecting the compound microscope with the Dino eye device [13]. Data of colony growth rate, conidia length, and width were statistically analyzed using SAS 9.4 software (SAS Institute, Cary, NC, USA) and means were compared using Tukey’s Studentized Range test (*p* ≤ 0.05).

### 2.3. Pathogenicity Assay

To assess virulence assay, an experiment was conducted in BSRI Ishurdi farm during the cropping season of 2020. Forty-one *C. falcatum* isolates were grown on oat meal agar (OMA) medium at 28 ± 1 °C for 15 days for good sporulations. The conidia of each isolates were harvested in sterilized distilled water, mixed, and adjusted to a concentration of 10^6^ conidia/mL for inoculation. Ten canes of Isd 28 variety were inoculated by the inocula of individual isolates at eight month old sugarcane plants using hypodermic syringe method [3,24]. Boring was done in the centre of the third inter node above the soil level of standing canes with the aid of a specially made inoculating borer (2 cm long and 2 mm diameter) up to the pith region for suitable inoculation and 0.1 mL of spore suspension (10^6^ conidia/mL) was inserted in the hole of each sugarcane plant employing a syringe. The punctured point was sealed with an insulating tape to prevent infection and oxidation. The inoculated canes were examined by cutting the cane longitudinally after two months of inoculation, following the symptomatic method [24,25,26] and graded based on an international scale 0–9 [27] as described in Supplementary Table S1. Disease reactions were categorized as R: resistant (0–2), MR: moderately resistant (2.1–4.0), MS: moderately susceptible (4.1–6.0), S: susceptible (6.1–8.0) and HS: highly susceptible (8.1–9.0) [24,25]. To assess pathogenic variation amongst the isolates, the same reactions were categorized as LeV: least virulent (0–2.0), LV: less virulent (2.1–4.0), MV: moderately virulent (4.1–6.0), V: virulent (6.1–8.0) and HV: highly virulent (8.1–9.0) [28]. Finally, the pathogenic reactions were grouped as less virulent (LeV and LV), moderately (MV) and virulent (V and HV) for group comparison [29]. At the end of each season, the respective isolates were re-isolated from infected cane tissues; sub cultured and kept at 4 ± 1 °C for future use.

### 2.4. Extraction and Purification of Genomic DNA

Genomic DNA was extracted using the technique described by Lin et al. [30] with minor modifications. Mycelial mats of each of the 41 isolates of *C. falcatum* were harvested from an eight day old culture grown on PDA. Fungal mycelia (150 mg) was ground by the adding of 600 µL of extraction buffer (50 mM EDTA, 250 mM NaCl, 100 mM Tris-HCl) using sterile mortar and pestle at room temperature. The macerated mycelia were transferred to a sterile 1.5 mL centrifuge tube, followed by the adding 60 µL of 60 µL of sodium dodecyl sulphate (2% SDS) and 12 µL ß-mercaptoethanol; it was then vortexed briefly to homogenize the mixture. The resultant suspension was placed in a water bath at 65 °C for 20 min. Afterwards 250 μL phenol 1:1chloroformwas added and mixed gently followed by centrifugation at 11,000 rpm for 10 min. The supernatant was transferred into a new sterile tube. Afterwards, 250 μL phenol 1:1chloroform was added and centrifuged at 11,000 rpm for 10 min. Thereafter, the aqueous layer (300 µL) was transferred into a new centrifuge tube and DNA was precipitated by adding an equal volume of ice-cold iso-propanol. The samples were incubated overnight at −20 °C to increase the DNA yield. Thereafter, DNA was centrifuged at 11,000 rpm for 10 min at 4 °C. The aqueous phase was drained and the DNA pellet was rinsed with 500 μL of 70% ethanol and air-dried after which it was suspended in a 50 μL of double distilled water (ddH_2_O). The purified DNA was quantified using Multiskan TM GO Microplate Spectrophotometer (Thermo Scientific^TM^, Hercules, CA, USA) at wavelengths (A260/A280 nm) and the ultimate strength of the DNA template was adjusted within 20–25 ng/μL.

### 2.5. PCR Amplification and Genesequencing

PCR amplification of fourgenes namely ITS- rDNA region, three protein-coding genes *β-tubulin* (Bt), Actin (ACT) and glyceraldehyde-3-phosphate dehydrogenase (GAPDH) of all *C. falcatum* isolates were subjected to amplification by fungal-specific primers ITS1(CTTGGTCATTTAGAGGAAGTAA), ITS4 (TCCTCCGCTTATTGATATGC), [31,32], Bt-1 (AACATGCGTGAGATTGTAAGT), Bt-2 (ACCCTCAGTGTAGT GACCCTTGGC) [33], ACT-512 (ATGTGCAAGGCCGGTTTCGC), ACT-783 (TACGAGTCCTTCTGGCCCAT) [34] and GDF1 (GCCGTCAACGACCCCTTCATTGA), GDR1 (GGGTGGAGTCGTACTTGAGCATGT) [35], respectively. The PCR reaction was conducted with a volume of 25 μL that had 12.5 µL MyTaq Red Mix, 2x (Bioline, Edge Business Centre, Humber Rd, London, UK), 1 µL DNA template (20–50 ng), 1 µL (10 µM) of each primer (forward and reverse) and 9.5 µL of free-nuclease water. PCR amplification was performed using a programmable thermal cycler (Eppendorf, Model:pro S, Hamburg, Germany) as follows: initial denature at 95 °C for 4 min, followed by 35 cycles of denaturation at 95 °C for 45 s; 45 s at the primer definite annealing temperature; extension at 72 °C for 1 min; and final extension at 72 °C for 10 min. The amplified products of PCR were detected by staining with florosafe DNA stain (Base Asia, Queenstown, Singapore) on 1.5% agarose electrophoresis gels in (1X) TBE buffer and visualized under UV transilluminator (Bio-Rad Laboratories Inc., Hercules, CA, USA). DNA fragments were sequenced in both directions with the predicted size of the ITS-rDNA region, *β-tubulin*, actin and GAPDH genes for all *C. falcatum* isolates by First BASE Laboratories Sdn. Bhd., Serdang, Selangor, Malaysia.

### 2.6. Alignment of Genesequence and Phylogenetic Analysis

The sequence results were verified using Sequence Scanner Software version 2.0 and aligned using BioEdit software version 7.0.90 [36,37]. Afterwards, the obtained consensus sequences were blasted in NCBI GenBank and checked the closed sequences similarity to the strains in the GenBank database. All sequences of *C. falcatum* isolates were deposited in the NCBI GenBank database. The history of evolutionary relationships phylogeny was constructed using maximum likelihood (ML) with 1000 bootstrap replication in molecular evolutionary genetic analysis version 7 (MEGA 7) [38]. Phylogenetic analyses wereconstructed according to the Kimura’ 2 parameter model [39], which included partial deletion of gaps/missing data collection and site coverage cut-off by 90%.The DNA sequence of *Monilochate infuscans* was assigned as an out-group.

### 2.7. ISSR-PCR Amplification

ISSR markers reported as highly polymorphic according to the previous studies were selected in this study. These markers were synthesized at the Apical Scientific Sdn. Bhd., Selangor, Malaysia (Table 2). The ISSR PCR reaction was carried out with a total volume of 25 μL comprising of 12.5 μL MyTaq Red Mix, 2x (Bioline, Edge Business Centre, Humber Rd, London, UK), 1 μL 10 μM primer, 1 μL DNA templates and 10.5 μL free nuclease water. The optimal temperature for the annealing was calculated for each primer. A Thermal cycler (Eppendorf, Model:pro S, Hamburg, Germany) was used to conduct all the PCR amplifications. PCR reaction was as follows: initial denaturation at 94 °C for 4 min, followed by 35 cycles of denaturation at 94 °C for 1 min, annealing temperature ranging from 43.9 to 61.6 °C for 1 min depending on the primer (Table 2), extension at 72 °C for 2 min and final extension at 72 °C for 10 min. PCR was repeated twice to ensure the consistency of the banding pattern. The amplified products of PCR were detected by staining with florosafe DNA stain (Base Asia, Queenstown, Singapore) on 2% agarose electrophoresis gels in (1X) TBE buffer and visualized under UV transilluminator (Bio-Rad Laboratories Inc., Hercules, CA, USA).

**Table 2:** Details of ISSR primers used in this study to differentiate *C. falcatum* isolates.

Primers	Sequences (5′–3′)	Primer Length (bp)	Annealing Temperature (°C)	References
ISSR-10	CACCACCACCACCAC	15	61.6	Gupta et al. [40]
UBC-825	ACACACACACACACACT	17	51.4	Arade et al. [41]
UBC-857	ACACACACACACACACYG	18	54.3	Arade et al. [41]
UBC-873	GACAGACAGACAGACA	16	43.9	Arade et al. [41]
ISSR845	CTCTCTCTCTCTCTCTRG	18	52.5	Patel et al. [1]
UBC810	GAGAGAGAGAGAGAGAT	17	49.0	Kaewchai et al. [42]
UBC828	TGTGTGTGTGTGTGTGA	17	53.0	Kaewchaiet al. [42]
UBC850	GTGTGTGTGTGTGTGTYC	18	54.0	Kaewchai et al. [42]
UBC860	TGTGTGTGTGTGTGTGGA	18	51.0	Soytong & Kaewchai [43]
ISSR 7	GGGCGAGAGAGAGAGAGAGA	20	44.0	Kaewchai et al. [42]

N.B. ‘Mixed bases for degenerate primers’ Y = C/T; R = A/T.UBC: ISSR Primers, designed from the University of British Columbia, Vancouver, Canada.

### 2.8. ISSR Data Analysis

The amplified DNA fragment sizes were assessed using UVIDocversion 99.02(Thermo Fisher Scientific, Waltham, MA, USA) compared to the 1 kb DNA ladder. The robust amplified DNA fragments were ranked 1 for the presence of DNA bands, and 0 for the absence of DNA bands. The scores were used to make a detailed matrix to assess genetic interactions. The ISSR-PCR product profiles of all isolates were referred to as cluster analysis to construct a dendrogram by unweighted pairs group method of the arithmetic mean (UPGMA) based on similarity coefficient of Jaccard using NTSYSPC, version 2.02 (Exeter Software, New York, NY, USA) [44]. The principal coordinates analysis (PCoA) was conducted to separate isolates by using GenAlEx version 6.50Exx [45].

The genetic structure parameters of total loci, number of polymorphism loci, polymorphism percentage, Shannon’s information index (I), Nei’s gene diversity (H) [46], the effective number of alleles (Ne) Kimura and Crow [47] and observed number of alleles (Na), were analyzed using population genetic analysis (POPGENE) 1.32 version [48]. The genetic distance between populations (D) was calculated by GenAlEx version 6.50Exx [39] from allele frequencies using the Nei [46] unbiased genetic distance. The genetic identity (I_N_) was determined using the I_N_ = 1-D formula. The coefficient of genetic differentiation (G*_ST_*) was calculated as G*_ST_* = (*H_T_* − *H_S_*)/*H_T_*, where *H_T_* corresponds to the total diversity of gene, and *H_S_* corresponds to the diversity of gene within sub-populations. Gene flows among populations (Nm) were measured using the formula: Nm = 0.5 (1 − G*_ST_*)/G*_ST_* [49]. If Nm < 1, the population continues to differentiate; if Nm ≥ 1, there will be a tiny distinction between populations, and migration is more important than genetic drift [44,50]. To hierarchically partition genetic variation of isolates within and between populations, the analysis of molecular Variation (AMOVA) was performed using Gen-AlExsoftware version 6.50 [45].

## 3. Results

### 3.1. Morphological and Colonies Variability

Visually, the isolates have distinct morphological characteristics of *C. falcatum*. Based on colonies and morphological features (growth pattern, colony colour, sporulation, and conidial size and shape), the 41 isolates were classified into two groups (C1-light type and C2 dark type). The isolates in group C1 have whitish-grey, greyish white mostly less fluffy, raised fluffy, and few flat colonies having medium to high sporulation. The isolates in group C2 have grey, dark grey mostly flat and raised fluffy colonies, and produced less to medium sporulation. The group C1 (the light type) consists of 21 isolates I-1, I-2, I-3, I-5, I-6, I-11, I-14, I-16, I-17, I-18, I-19, I-20, I-21, I-25, I-26, I-31, I-35, I-38, I-39, I-41, I-42 and group C2 (dark type) is made up the remaining 20 isolates I-7, I-8, I-9, I-10, I-12, I-13, I-15, I-22, I-23, I-24, I-27, I-28, I-29, I-30, I-32, I-33, I-34, I-36, I-37 and I-40 (Table 3).

**Figure 2:**
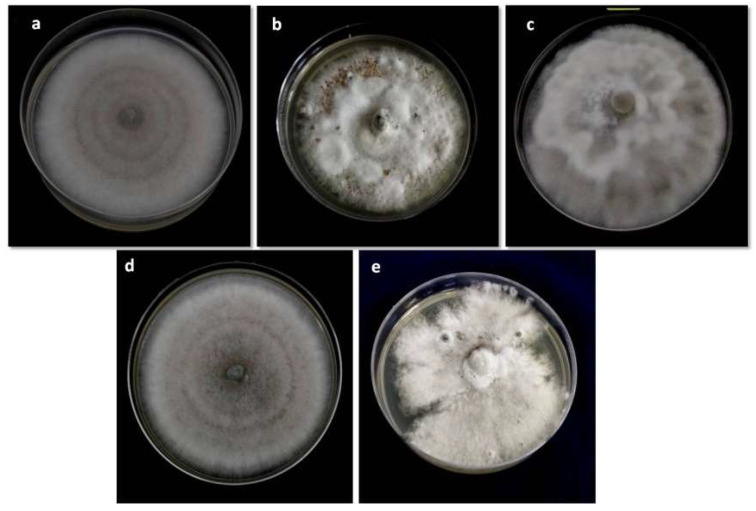
Colonies characteristics of *C. falcatum* based on topography type (**a**) Flat (**b**) Less fluffy (**c**) Raised fluffy; and margin type (**d**) Smooth (**e**) Irregular as observed in different isolates.

**Figure 3:**
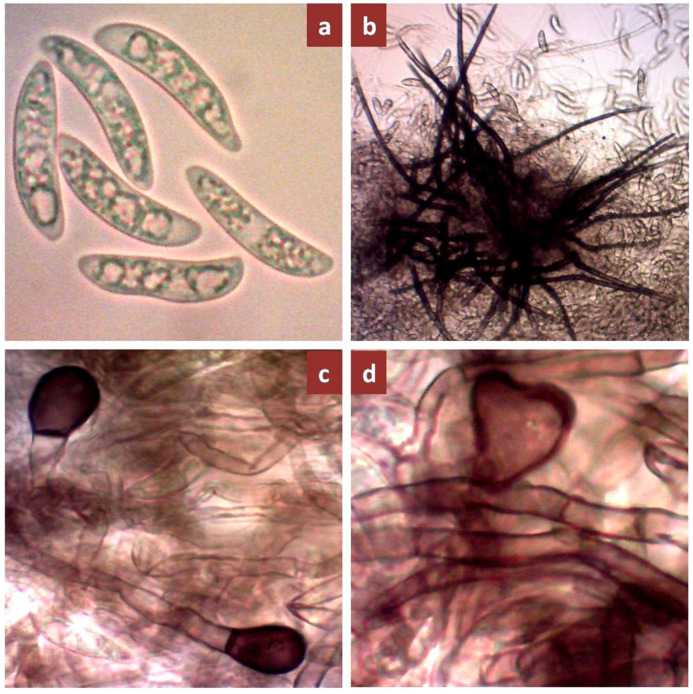
Microscopic features of *C. falcatum* isolates from Bangladesh (**a**) Conidia (**b**) Setae (**c**) Globose type appressoria (**d**) Clavate type appressoria.

In terms of mycelial growth rate, there were significant differences among the isolates. Growth rates of the mycelia ranged from 5.97 to 12.14 mm day^−1^; averaging 9.70 mm day^−1^ (Table 3). A Smooth and irregular mycelium margin was observed among the 41 isolates (Figure 2). All the isolates produced setae, globose or clavate-edged appressoria and aseptate hyaline one-cell, falcate or sickle-shaped conidia (Figure 3). Significant variations occurred in the mean length and width of the conidia. The conidial size of the isolates ranged from 20.86 to 33.51 μm in length and 5.33 to 8.46 μm in width, with an average of 26.98 × 6.41 μm for conidial length and width (Table 3).

### 3.2. Pathogenic Variability

**Figure 4:**
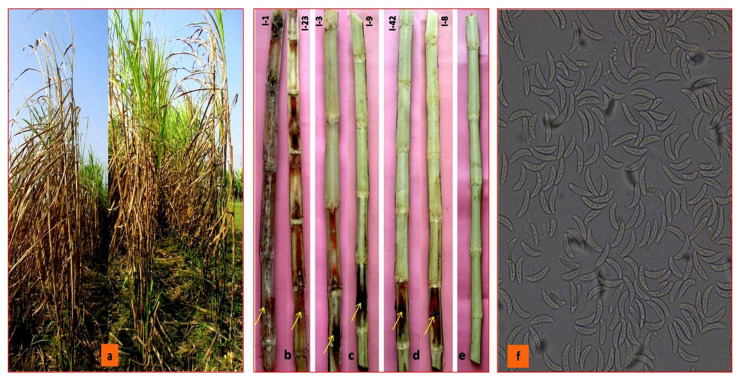
Typical symptoms of red rot disease on inoculated sugarcane variety, Isd 28 under field trial condition 60 days after inoculation. (**a**) Disease symptom from three virulence groups of 41 *C. falcatum* (**b**) severe internal symptom induced by virulent isolate (**c**) Moderate internal symptom induced moderately virulent isolate (**d**) mild internal symptom induced less virulent isolates (**e**) Un-inoculate cane, (**f**) Conidia of *C. falcatum* under compound microscopes (100×) after re-isolation from red rot infected cane tissue. The arrow indicated the point of inoculation.

The artificial inoculation of *C. falcatum* isolates on sugarcane variety, Isd 28 induced red rot disease symptoms under field conditions (Figure 4a). Out of 41 *C. falcatum* isolates, eight isolates produced highly susceptible (HS), two susceptible (S), seven moderately susceptible (MS), 18 moderately resistant (MR), and six resistant (R) reactions to sugarcane variety Isd 28 (Table 4). The virulence level of the 41 *C. falcatum* isolates corresponding to disease reaction on host plant were categorized into three groups: virulent, moderately virulent, and less virulent (Figure 4b–d). The significant virulence variation was observed among the *C. falcatum* isolates from four regions of Bangladesh, three types of virulence levels were recorded among the isolates in Rajshahi and Khulna regions and out of the 17 isolates in Rajshahi, seven were virulent (41.18%), three were moderately virulent (17.64%), and seven were less virulent (41.18%). Out of the11 isolates in Khulna, one isolate was virulent (9.09%), two isolates were moderately virulent, (18.18%) and eight isolates were less virulent (72.72%). In addition, the isolates from Rangpur and Dhaka regions were moderately virulent and less virulent types. In the Rangpur region, two out of the four isolates were moderately virulent (50%) and the rest of the two isolates were less virulent (50%). In the Dhaka region, out of the nine isolates, two were moderately virulent (22.22%) and seven isolates were less virulent (77.78%) (Table 4). Re-isolation of all isolates produced typical falcate conidia under the compound microscope. It was confirmed as *C. falcatum* (Figure 4f).

### 3.3. Molecular Identification of C. falcatum Isolates

The final sequences of the four genes for the isolates were identified as *C. falcatum* (570 bp ITS-5.8S rDNA, 750 bp β-tubulin, 271 bp ACT, and 150 bp GAPDH). The sequences of the four genes of *C. falcatum* isolates were deposited in the NCBI database and it revealed 99% similarity to conserved gene sequences (Supplementary Table S1). Alignment of the ITS rDNA, β-tubulin, ACT, and GAPDH sequences was compared to the sequences in the GenBank database to confirm the identity of the fungus. It revealed 99%similarity to the ITS-5.8S rDNA sequence of the published *C. falcatum* isolate (Cf01) with GenBank accession number KU220959. It also demonstrated 99% consistency with the *β*-*tubulin* sequences of *C. falcatum* isolates LC885 (JQ00586), followed by 99% consistency with the ACT sequence of *C. falcatum* isolates LC885 (HM171665), and 99% consistency with the GAPDH sequence of *C. falcatum* isolates LC885 (HM171671).

### 3.4. Phylogenetic Analysis of ITS-rDNA Region

**Figure 5:**
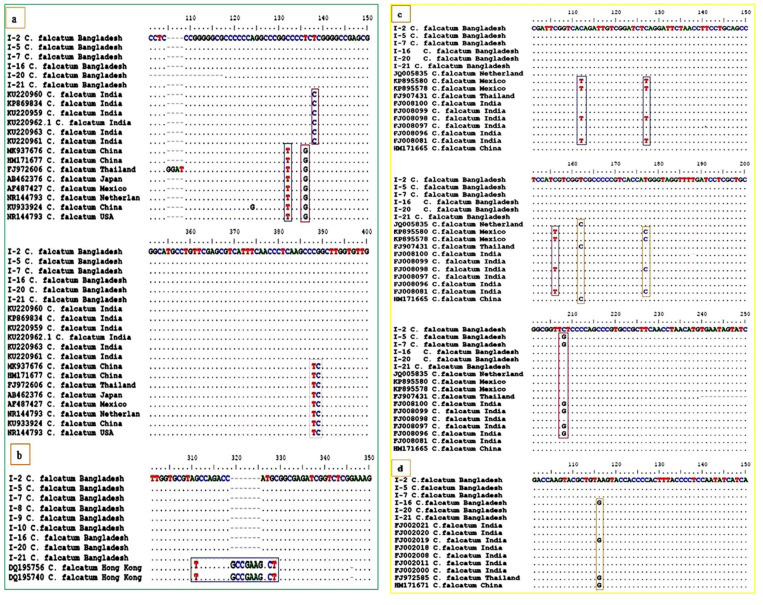
Nucleotides variation of *C. falcatum* isolates from Bangladesh compare with other countries isolates at different loci positions using (**a**) ITS (**b**) β-*tubulin* gene (**c**) Actin and (**d**) GADHP gene sequences.

ITS sequences of *C. falcatum* from India, Thailand, China, Japan, Netherlands, Mexico, USA, and *C. endophytum, C. gloesporium, C. acutatum* species complex were analyzed with *C. falcatum* in this study dataset to determine the phylogenetic relationship of the Bangladesh *C. falcatum* isolates. The final alignment of ITS sequences consisted of 63 taxa, including 41 *C. falcatum* isolates from this present study, and 22 reference sequences from the GenBank. The phylogenetic tree generated by Maximum likelihood analysis gave strong support in all clades with high bootstrap values indicated at the nodes. *Colletotrichum falcatum* isolates were divided into three clades: clade I (Bangladesh), clade II (India), and clade III (other countries) using ITS phylogram. The isolates from Bangladesh were included in clade I, and there was no regional trend in the spread of the isolates within this clade. Clade II consisted of the Indian isolates and they had no regional structure within the clade. Bangladesh and Indian clades were rebuilt as sister clades. Clade III composed of three Asian (China, Japan and Thailand), two American (USA and Mexico) and one European (Netherlands) *C. falcatum* isolates, respectively (Supplementary Figure S2a). The ITS rDNA sequences revealed that *C. falcatum* isolates from Bangladesh differed from *C. falcatum* isolates from other countries (India, China, Thailand, Mexico, USA, Japan and Netherlands) by substitution in five loci at positions:132,136, 138, 388 and 389 (T/G/C/TC), respectively (Figure 5a). Other *Colletotrichum* species were clustered into distinct clades with a high distance from the *C. falcatum* isolates.

### 3.5. Phylogenetic Analysis of β-Tubulin Gene Region

The final alignment of the *β*-*tubulin* sequences revealed 51 taxa including 41 *C. falcatum* isolates from this present study and 10 *Colletotrichum* species of reference sequences in the GenBank. The Maximum likelihood phylogenetic analysis produced strong support for the clades (high bootstrap value). The phylogram of the *β*-*tubulin* gene suggested that *C. falcatum* isolates were separated into two clades: clade I (Bangladesh), clade II (Hong Kong). Clade I consisted of the isolates from Bangladesh and there was no geographic structure in the distribution of the isolates within this clade. The clade II composed of Hong Kong isolates (Supplementary Figure S2b). The sequences of the *β*-*tubulin* suggest that *C. falcatum* isolates from Bangladesh differed from *C. falcatum* isolates from Hong Kong through substitution in three loci [(311, 327 and 328 (T/C/T) (Figure 5b)]. Other *Colletotrichum* species were clustered in other clades with a high distance from the *C. falcatum* isolates.

### 3.6. Phylogenetic Analysis of Actin Gene Region

The final sequence alignment of the Actin consisted of 61 taxa, including 41 *C. falcatum* isolates from the present study and 20 *Colletotrichum* species of reference sequences in the GenBank. The Maximum likelihood phylogenetic analysis generated strong support for the clades with high bootstrap values. The Actin gene, such as the ITS, divided the isolates into three separate clades. Clade I consisted of the isolates from Bangladesh and four isolates from India. Whereas, clade II consisted of three isolates from Thailand, Netherlands, and China. Clade I and II were reconstructed as sister clades. Clade III was made up of two sequences of *C. falcatum* from Asia (India) and another three from America (Mexico) (Supplementary Figure S3a). The actin gene sequences revealed that *C. falcatum* isolates from Bangladesh differed from *C. falcatum* isolates from other countries such as India, China, Thailand, Mexico and Netherlands by substitution in six loci at positions: 113, 127, 156, 162, 177 and 208 (T/T/T/C/C/G) (Figure 5c). Other *Colletotrichum* species clustered in distinct clades from the *C. falcatum* isolates.

### 3.7. Phylogenetic Analysis of GAPDH Gene Region

The GAPDH gene alignment comprises sequences from 58 isolates including 41 *C. falcatum* isolates from this study and 17 (*C. falcatum* and other species) reference sequences in the GenBank. The GAPDH gene phylogram separated the Bangladesh isolates including those from India, China, and Thailand in a clade with high bootstrap values. Two Bangladesh isolates, one China, and one Thailand isolates differed from other Bangladesh and India sequences, although they belong to the same clade (Supplementary Figure S3b). There were no significant differences among Bangladesh, India, China, and Thailand sequences. GAPDH gene sequences of America and Europe isolates were not available in the NCBI database. The GAPDH sequences revealed that *C. falcatum* isolates from Bangladesh differed from *C. falcatum* isolates from India, China, and Thailand by substituting in loci at positions 116 (G) (Figure 5d). Other *Colletotrichum* species were clustered into clades with a high distance from the *C. falcatum* isolate.

### 3.8. Phylogenetic Analysis of Combined Gene Regions

**Figure 6:**
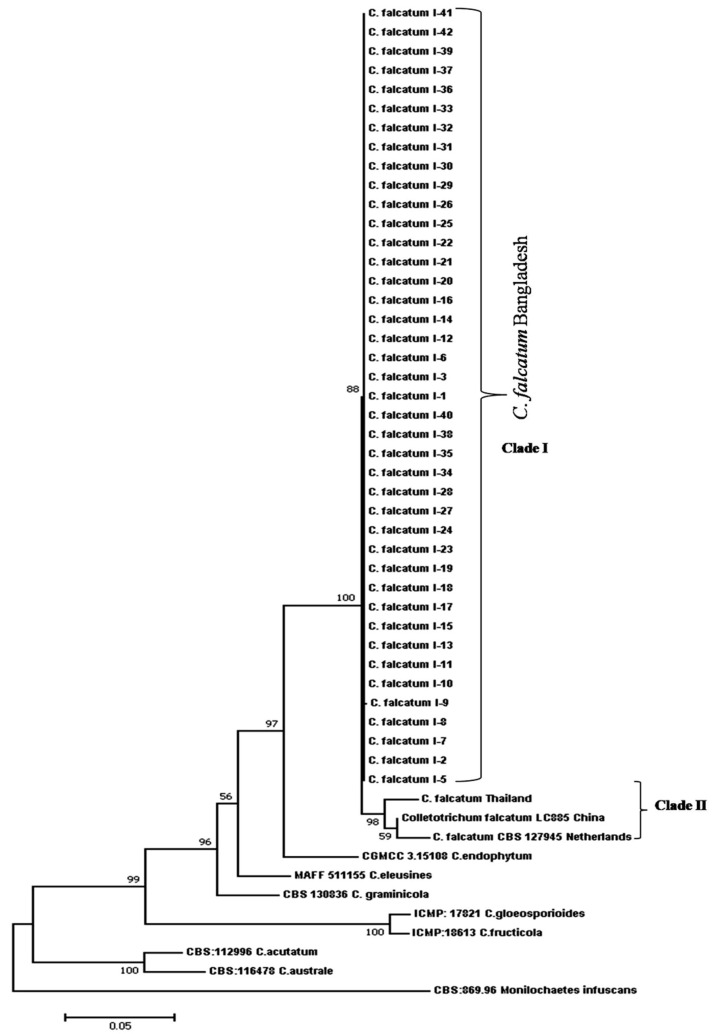
Phylogenetic tree of *C. falcatum* isolates using Maximum likelihood method based on combined ITS, *β-tubulin*, Actin and GAPDH sequences. The tree was rooted with *Monilochaetesinfuscan*. ML bootstrap value is indicated at each node.

The final combined dataset of ITS, *ß-tubulin*, actin, and GAPDH had 1777 characters after alignment and consisted of 52 isolates including 41 *C. falcatum* isolates from this study and 10 (3 *C. falcatum* and 7 other *Colletotrichum* species) reference sequences in the GenBank. The *C. falcatum* isolates were identified and confirmed using sequence data of ITS, *ß-tubulin,* actin, and GAPDH. The Maximum likelihood phylogenetic analysis produced strong support for the clades with high bootstrap values. The phylogram of the combined four genes revealed that *C. falcatum* isolates were divided into two clades: clade I (Bangladesh) and clade II (Other countries). Clade I consisted of *C. falcatum* isolates from Bangladesh and there was no geographical structure in the distribution of the isolates within the clade. Clade II is composed of Thailand, China, and Netherlands *C. falcatum* isolates (Figure 6). Thailand *C. falcatum* isolates differed from China and Netherlands, although they clustered in the same clade. Bangladesh and other countries clades were reconstructed as sister clades. The same strains of four genes sequences of American and Indian *C. falcatum* isolates were not available in the NCBI database. Combined four genes sequences also separated different species of *Colletotrichum* to various distinguished clades with a high distance from *C. falcatum.* Our findings revealed that *C. endophytum* was the closest species of *C. falcatum* which indicated the closest genetic background to *C. falcatum* isolates worldwide (Figure 6).

### 3.9. ISSR Analysis

#### 3.9.1. Genetic Diversity

**Figure 7:**
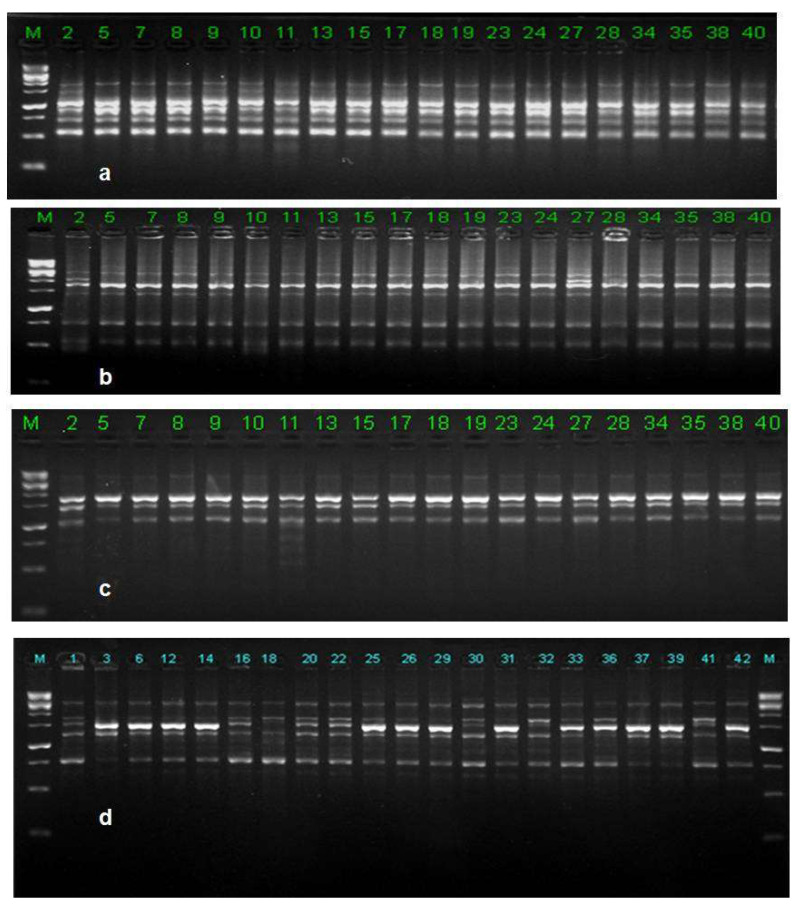
Representative DNA fingerprinting profiles of *C. falcatum* isolates generated by different ISSR molecular markers (**a**) UBC 810; (**b**) ISSR 10; (**c**) UBC 860 and (**d**) UBC 873.

Forty-one *C. falcatum* isolates were subjected to ISSR markers to demonstrate a reasonable polymorphism (Figure 7). ISSR analysis of 10 selected markers were revealed based on polymorphic banding patterns and generated 404 bands, which varied from 210–2770 bp in size of which 400 were polymorphic. Maximum number of bands were recorded by the marker UBC-873 (46 bands), whereas the minimum number was generated by the marker with UBC810 (37 bands). ISSR markers polymorphic percentage ranged from 94.59% to 100% with a mean of 99.01%, whereas the number of polymorphic bands among 10 markers ranged from 35 to 45 loci (Table 5). The observed number of alleles (Na) was 1.9901, the effective number of alleles (Ne) was 1.2425, Nei’s gene diversity (H) was 0.1732 and Shannon index of diversity (I) was 0.2985 at the species level (Table 6). Genetic variation was slightly lower at the population level than at the species level.

At the population level, the percentage of polymorphic bands (PPB) ranged from 34.16 to 68.81% with a mean of 53.40%. The average number of alleles (Na) was 1.5341; the effective number of alleles (Ne) was 1.2370; Neil’s gene diversity (H) 0.1521 and Shannon’s Information Index (I) was 0.2396 (Table 6). The order of genetic diversity among the population levels based on Nei’s gene diversity and Shannon’s Information Index was in the order of Ran < Khu < Dha < Raj (Table 6).

#### 3.9.2. Populations Structure

**Figure 8:**
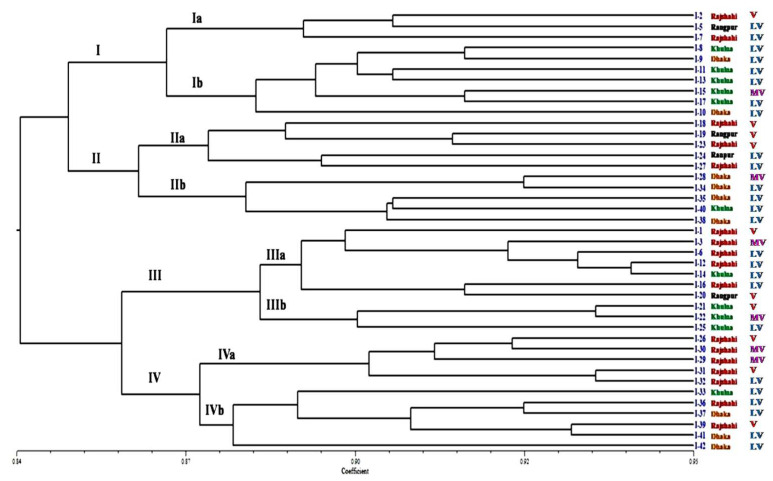
Dendrogram derived from UPGMA cluster showing the genetic relationships among the 41 *C. falcatum* isolates from four regions of Bangladesh. Note: Virulence level denoted as V = Virulent; MV = Moderately virulent and LV = Less Virulent.

Using Nei’s gene diversity statistics, the genetic structure was explored further. The analysis of the occurrence of genetic variation revealed that mean the gene diversity within populations (*Hs*) and total gene diversity (H*_T_*) were 0.152 and 0.173, respectively (Table 6). The genetic diversity coefficient among the populations (G*_ST_*) was 0.122. The G*_ST_* value less than 1 suggests high level of genetic similarity and low degree of genetic diversity among the populations. The high level of genetic similarity in the population influenced the high level of gene flow among the population. Gene flow (*Nm*) among the populations was 3.584. The results of AMOVA revealed that 78% of the total variation occurred within the population isolates, whereas 22% of the variability was attributable to the difference among the populations (Table 7). A high degree of genetic identity occurred in Rajshahi (Raj) and Dhaka (Dha) populations. In contrasts, a comparatively low degree of genetic identity was observed in Khulna (Khu) and Dhaka (Dha) populations, with estimated identities of 0.991 and 0.970, respectively (Table 8). Based on ISSR marker data, cluster analysis was carried out to construct a dendrogram using UPGMA. The results revealed genetic associations in the populations (Figure 6). The highest value of cophenetic correlation (r = 0.919) was determined by the Jaccard similarity coefficient, suggesting a very strong match between the similarity coefficient and the clustering method. To estimate the genetic relationships between isolates, the UPGMA cluster analysis of Jaccard’s similarity coefficients generated a dendrogram that categorized the 41 *C. falcatum* isolates into four major clusters (Figure 8). Cluster I consisted of 10 isolates (I-2, I-5, I-7, I-8, I-9, I-10, I-11, I-13, I-15, and I-17). Cluster II consisted of 10 isolates (I-18, I-19, I-23, I-24, I-27, I-28, I-34, I-35, I-38, and I-40). Cluster III consisted of 10 isolates (I-1, I-3, I-6, I-12, I-14, I-16, I-20, I-21, I-22, and I-25).

**Figure 9:**
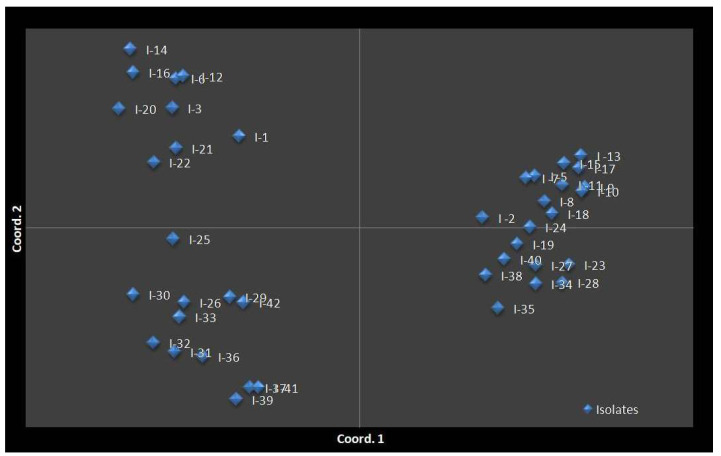
Two-dimensional principal coordinate analysis (PCoA) showing the pattern of sub-clustering of 41 isolates of *C. falcatum* isolates from four regions of Bangladesh.

However, cluster IV consisted of 11 isolates (I-26, I-29, I-30, I-31, I-32, I-33, I-36, I-37, I-39, I-41, and I-42). Each cluster had two sub-clusters. The results suggest that the *C. falcatum* isolates collected from the same location/region belong to the same sub-cluster as noted in the clusters Ib, IIb, IIIa, and IVa. However, few of them belong to other sub-clusters/cluster indicating they are partially related to the geographical regions because of gene flow and planting materials exchanged from one region to another. Furthermore, the findings revealed that, genetic diversity correlated weakly with the virulence level of the tested isolates because the MV and V isolates had formed no specific cluster in the phylogenetic tree. PCoA results were consistent with those with cluster analysis. The PCoA results supported clustering into the four population groups (Figure 9).

## 4. Discussion

*Colletotrichum falcatum* causes red rot disease in sugarcane. This disease is transmitted from one location to another by infected sugarcane [51]. Several measures have been developed to effectively control the disease, but the measures have not successful. One of the major reasons is due to fact the pathogen develops new races to easily infect existing sugarcane cultivars [9]. Thus, many popular sugarcane varieties such asIsd 17, Isd 18, Isd 28 and Isd 32, have been withdrawn from commercial fields in Bangladesh [52]. This is the first study to determine the genetic variation, population structure of *C. falcatum* isolates and their race prevalence in Bangladesh. Forty-one *C. falcatum* isolates were collected from different districts of the four regions in Bangladesh after which their morphological, virulence, phylogenic relationship and genetic diversity were determined.

Morphological and colony differences indicated that there were significant differences among the isolates. The isolates with whitish-greyand greyish-white colony colour were mostly fluffy; few of them possess flat topography and they produced medium to high sporulation. The grey and dark grey isolates were mostly flat, although some demonstrated raised fluffy and produced low to medium sporulation. The findings are comparable to those of Kaur et al. [23], who also reported that the isolates had a whitish grey and greyish white color colony which is mostly fluffy and produces medium to high sporulation and the colonies were grey, dark grey, and less fluffy. In a related study, Prema et al. [22] reported significant difference in the morphology and cultural characteristics of *C. falcatum*. The 41 isolates demonstrated difference in mycelial growth rate and these findings are consistent with that of Viswanathan et al. [53] who focused on nine main pathotypes of *C. falcatum*. In terms of conidial morphology, the 41 isolates produced falcate shape; hyaline conidia and the conidial size were similar to the results of Mishra and Behera [54], Sangdit et al. [21] and Prema et al. [22]. Although, *C. falcatum* isolates are grouped based on colony and morphological characters, these approaches are not reliable or consistent because of the fungal colony and morphological characters are affected by environmental factors. Moreover, morphological data of *C. falcatum* such as growth rate and sporulation correlate weakly with the frequency of the disease infection on sugarcane [55,56]. The Pathological assessment revealed that the *C. falcatum* isolates induced red rot disease symptoms on sugarcane variety, Isd 28 through artificial inoculation with different severity. Findings revealed that, they were clustered into three virulence cluster namely LV(less virulent), MV (moderately virulent) and V (virulent). The isolates from MV and LV clusters are widely spread in the four major sugarcane growing regions in Bangladesh. However, the isolates from Vcluster are mostly distributed in the sugar mill zone. *Colletotrichum falcatum* belongs to an anamorphic fungus and because of this, the virulence variability within populations might have occurred through mutation, heterokaryosis, hybridization and adaption [55]. Other possible reasons could be variation in the production of hydrolytic enzymes (pectinolytic and cellulolytic) and melanin during host-pathogen interaction. In Bangladesh, susceptible sugarcane varieties/cultivars are widely planted by smallholder. This enables the pathogen to evolve and diversify its virulence because of the high proliferation rate and dispersion capacity. Cluster analysis revealed that there was a weak correlation between virulence level and genetic diversity amongst *C. falcatum* isolates (Table 4 and Figure 8). This finding corroborates the previous reports pertaining the virulence level of pathogens isolated from sugarcane and geographical distribution [23,28,57].

The final sequence of ITS, *β–tubulin*, actin, and GAPDH genes were identified as *C. falcatum* and revealed high similarity to the reference sequences in the Genbank database. These results are consistent with the findings reported by Sangdit et al. [21] for molecular identification of *C. falcatum*, Mahmodi et al. [58] and Aktaruzzaman et al. [59] for *C. tuncatum*. Sequences of the ITS, *β-tubulin,* actin, and GAPDH have been widely used to determine the phylogenetic relationships among many *Colletotrichum* species which had clarified the taxonomic relationships within the genus [51,60]. In this present study, ITS, *β-tubulin,* actin, and GAPDH genes sequences data were used in combination with the first time for phylogenetic analysis of *C. falcatum* that contributed to the taxonomic study in Bangladesh and the world as a whole. Furthermore, the accuracy of molecular identification is high and more reliable compared with the conventional methods to especially classify different genus of *Colletotrichum* which are closely related species [14,61,62]. The traditional method is less successful due to the assumed presence of intermediate forms between species, morphologic plasticity, and overlapping phenotypic characters. In contrast the molecular biology technique encompasses alternative and supplementary methods for overcoming the difficulties in identifying up to species level [63]. The molecular method is reputed for enabling rapid identification of isolates and it also clarifies the relationships between fungal organisms [64].

The genetic relatedness and ancestry of *C. falcatum* were determined and the four examined genes used were successfully differentiated. The collection of molecular entries of *C. falcatum* in the NCBI database were relatively limited. Thus, selected *C. falcatum* isolates were compared to Bangladesh isolates to determine the genetic relationship of *C. falcatum* isolates worldwide. The comparison was carried out based on the ITS, *β-tubulin,* actin, and GAPDH sequence data to identify the phylogenetic relationship of *C. falcatum* isolates. Evolutionary history through maximum likelihoodtree based on four genes inferred that there was no geographical structure in the distribution of *C. falcatum* isolates among the different sites in Bangladesh. Four gene sequences also revealed that *C. falcatum* isolates from Bangladesh differed from reference *C. falcatum* isolates from other countries (India, China, Thailand, Mexico, USA, Japan, and the Netherlands) because of the variation of nucleotide in different loci positions. The Actin and GAPDH genes phylogenetic analysis showed that several *C. falcatum* isolates from India, one from China, and one from Thailand, were cluster together with the Bangladesh isolatesThis is related to the fact that: (i) India, China, and Thailand are geographically close to Bangladesh and (ii) Planting materials are exchanged among Southeast Asian countries including India, China, and Thailand. This assumption is supported by the findings of Oghenekaro et al. [65]. Four genes phylogenetic tree comparison results demonstrated that *C. endophytum* was the closest species to *C. falcatum* and this observation is consistent with that of Hyde et al. [66], who stated that *C. endophytum* is a sister taxon of *C. falcatum*.

ISSR-PCR fingerprinting is a valuable method for population structure studies and differentiating individual fungal isolates [67]. ISSR-PCR is more accurate than the RAPD-PCR variant for determining the genetic variation of the individual isolates [68,69]. In this present study, 10 selected ISSR markers were used in the genetic diversity analysis against 41 *C. falcatum* isolates. These ISSR markers gave different reproducible bands, and the high percentage polymorphism suggests genetic variation among the isolates. The different band pattern generated by ISSR markers in the study could be attributed to (i) intraspecific variation: ISSR fingerprinting is higher for certain fungal species and lower for others because of intraspecific variations among the markers [60],(ii) abundant in (CA)n and (GT)n content: this may clarify the more significant number of alleles reported within those loci and the more polymorphic ISSR-PCR bands [69], (iii) primer-binding site: absence of a primer-binding site due to mutationin priming site, strand slippage during DNA polymerization due to template instability, deletions or insertions that might decrease orincrease the amplifiedfragment length sufficiently to be scored as a separate locus, and structural rearrangements of the chromosomes that prevent polymerization [70], and (iv) base composition: The base content of ISSR primers influence the quality of fingerprints, as primers having higher GC content (>50 percent) consequence in background noise and non-homologous co-migrating fragments, while pure A or T primers resulted in amplification of the products. Adjusting the annealing temperature of primer and other conditions of reaction reduces these effects [71].

The genetic diversity of *C. falcatum* was higher at the species level than at the population level in this study. Among the four populations of *C. falcatum*, different (highest to lowest) genetic diversity was evident. Variations of genetic diversity among the populations might have resulted from some factors. These factors are as follows: (i) population size: larger populations maintain greater genetic diversity under neutrality; (ii) age of population: Aged populations have a more extensive genetic diversity than the newly colonized habitat, mainly if only a few colonizers form this population. The older population could have mutational events to add new genetic variants and for genetic drift to increase the prevalence of these alleles to measurable levels; (iii) location: the isolates located at or near the center of origin from which the species originate have a higher degree of gene diversity than the isolates from other areas because the original population are older [72,73,74]. Weeds et al. [74] illustrated that the genetic variance in *C. gloeosporioides* isolates are higher where native or naturalized host species occur compared with areas where the host species have recently been introduced.

The evidence of admixture of some of the *C. falcatum* isolates from the four populations occurred in this present study. This occurred because of climatic conditions of these locations were similar. The similar climate enabled the isolates to co-exist. Other factors such as spontaneous natural mutations, genetic drift, gene flow, crop rotation, sampling year, and alternate host might have played significant role in terms of variation among the isolates of *C. falcatum* [70,75,76,77,78]. Furthermore, molecular markers are more informative than rDNA-ITS sequencing in terms of detecting the diversity within the individual population but less successful in detecting the variation between different populations [79].

## 5. Conclusions

Morphological identification is a primary method to obtain first-hand information and idea about pathogens. However, some ambiguities arise in identifying the red rot pathogen because of overlapping of their morphological characters. For this reason, the molecular approach is the best alternative methods used to overcome the problem in *C. falcatum* identification and characterization. The sequence analysis of ITS, *β-tubulin,* actin, and GAPDH genes region provides accurate information on the characterization and phylogenetic understanding of *C. falcatum* isolates from Bangladesh. This information enables plant pathologist to identify potential or new strains/pathotypes. The information gained from pathological and molecular diversity will lead to a better understanding and effective disease control of the *C. falcatum* in Bangladesh. The ISSR markers datasuggest that genetic diversity exists in the *C. falcatum* isolates. The results provide precautionary information for plant breeders and pathologists because of the continued evolution of *C. falcatum*. Joint efforts from all parties are required to breed new disease resistant sugarcane variety to effectively control red rot in the field. To the best of our knowledge, this present study has provided first hand information on phylogenetic analysis and genetic diversity of *C. falcatum* in Bangladesh.

## Figures and Tables

**Table 1:** Description of *C. falcatum* Went isolates collected from various sugarcane growing regions of Bangladesh used in this study.

SL No.	Isolates Code	Host Cultivar	Age of Plant (Month)	Site of Collection	Latitude (°N), Longitude (°E)	Name of Regions
1	I-1	Isd 18	7	BSRI, Ishurdi	24.1153°, 89.0817°	Rajshahi Region
2	I-2	Clone	8	BSRI, Ishurdi	24.1153°, 89.0817°	
3	I-3	Isd 18	7	BSRI, Ishurdi	24.1153°, 89.0817°	
4	I-6	BSRI Akh 42	8	NTSM, Natore	24.4102°, 89.0076°	
5	I-7	BSRI Akh 42	8	Bonpara, Natore	24.2942°, 89.0812°	
6	I-12	I-291-87	8	Chapainobabgonj	23.1657°, 89.4990°	
7	I-16	Q 69	7	Ranihati	24.6329°, 88.1929°	
8	I-18	Q 69	8	Ranihati	24.6329°, 88.1929°	
9	I-23	BSRI Akh 42	7	NTSM, Natore	24.4102°, 89.0076°	
10	I-26	Isd 18	8	BSRI, Ishurdi	24.1153°, 89.0817°	
11	I-27	Q 69	8	Chapainawabgonj	24.7413°, 88.2912°	
12	I-29	Isd 18	6	BSRI, Ishurdi	24.1153°, 89.0817°	
13	I-30	BSRI Akh 42	8	Bonpara	24.2942°, 89.0812°	
14	I-31	BSRI Akh 42	8	Bonbara	24.2942°, 89.0812°	
15	I-32	Isd 18	7	BSRI, Ishurdi	24.1153°, 89.0817°	
16	I-36	Isd 18	8	BSRI, Ishurdi	24.1153°, 89.0817°	
17	I-39	I 291-87	6	BSRI, Ishurdi	24.1153°, 89.0817°	
18	I-5	BSRI Akh 42	8	TSM	26.0504°, 88.4219°	Rangpur Region
19	I-19	Strains	8	Shampur	25.6969°, 89.1451°	
20	I-20	Clone	7	Shampur	25.6969°, 89.1451°	
21	I-24	Isd 16	11	TSM	26.0504°, 88.4219°	
22	I-8	Co 975	8	Patkelghata	22.7652°, 89.1623°	Khulna Region
23	I-13	Local Chewing	8	Chowgacha	23.2632°, 89.0203°	
24	I-14	Local Chewing	8	Sharsa (N)	23.0693°, 88.9605°	
25	I-15	Co-208	9	Narail	23.1657°, 89.4990°	
26	I-17	Isd 18	7	MKSM	23.4139°, 89.1333°	
27	I-21	Isd 18	8	Magura	23.4855°, 89.4198°	
28	I-22	Co 975	9	Patkelghata	22.7652°, 89.1623°	
29	I-25	Local Chewing	6	Jhikorgacha	23.0999°, 89.0991°	
30	I-33	Co 208	9	Narail	23.1657°, 89.4990°	
31	I-40	GT 17	7	Chuadanga	23.6161°, 88.8263°	
32	I-11	Isd 16	10	Barisal	22.7010°, 90.3535°	
33	I-9	Isd 16	10	Kalkini	23.0724°, 90.2808°	Dhaka Region
34	I-10	BSRI Akh 42	8	V-Mirzapur	24.0902°, 90.4073°	
35	I-28	Isd 18	7	Nokla	24.9753°, 90.2057°	
36	I-35	BSRI Akh 42	8	Manikgonj	23.8617°, 90.0003°	
37	I-37	BSRI Akh 42	9	Manikgonj	23.8617°, 90.0003°	
38	I-38	Local	8	Kalkini	23.0724°, 90.2808°	
39	I-41	BSRI Akh 42	9	TungiPara	22.9073°, 89.8985°	
40	I-42	BSRI Akh 42	10	Mirzapur	24.1053°, 90.1051°	
41	I-34	BSRI Akh 42	9	Bandarban	21.8311°, 92.3686°	

**Table 3:** Colonies and conidial characteristics of *C. falcatum* isolates from Bangladesh.

Isolates	Colony Colour	Topography	Margin	Colony Radial Growth Rate/Day (mm)	Conidia Length (µm) *	Conidia Width (µm) *	Sporulation
I-1	Whitish grey	Flat	Smooth	8.40 g–k	25.69 i–p	6.02 bcde	+++
I-2	Greyish white	Flat	Smooth	9.79 c–g	25.52 k–p	6.45 bcd	+++
I-3	Whitish grey	Less fluffy	Smooth	10.57 a–f	26.40 h–n	6.28 bcd	+++
I-5	Greyish white	Flat	Smooth	10.04 b–g	24.66 m–p	6.38 bcd	+++
I-6	Greyish white	Less fluffy	Irregular	6.95 k–m	25.17 l–p	6.394 bcd	+++
I-7	Grey white	Flat	Smooth	10.58 a–f	29.29 c–g	6.68 bcd	++
I-8	Dark grey	Flat	Smooth	10.63 a–e	28.40 c–j	6.67 bcd	++
I-9	Grey	Flat	Smooth	10.63 a–e	28.38 c–k	6.49 bcd	+
I-10	Grey	Raised fluffy	Smooth	10.63 a–e	27.27 f–m	6.43 bcd	++
I-11	Greyish white	Less fluffy	Smooth	9.02 e–i	24.01 n–p	6.71 bc	+++
I-12	Dark grey	Flat	Irregular	6.04 m	25.31 l–p	5.94 cde	++
I-13	Grey	Flat	Smooth	10.63 a–e	25.06 l–p	6.05 bcde	++
I-14	Greyish white	Raised fluffy	Smooth	7.31 j–m	25.55 j–p	6.39 bcd	++
I-15	Gray	Raised fluffy	Smooth	10.63 a–e	24.53 m–p	5.33 e	++
I-16	Greyish white	Flat	Irregular	5.97 m	27.57 e–k	6.19 bcde	+++
I-17	Greyish white	Flat	Irregular	9.71 c–h	25.05 l–p	6.77 bc	+++
I-18	Greyish white	Flat	Irregular	8.09 i–m	27.18 f–m	5.99 cde	+++
I-19	Greyish white	Flat	Irregular	9.42 c–h	25.76 i–p	6.40 bcd	+++
I-20	Greyish white	Raised fluffy	Irregular	11.54 ab	27.04 f–m	6.45 bcd	+++
I-21	Whitish grey	Raised fluffy	Smooth	10.95 a–d	26.78 f–n	5.94 cde	+++
I-22	Grey	Raised fluffy	Smooth	10.12 b–f	27.10 f–m	6.36 bcd	++
I-23	Dark grey	Flat	Smooth	10.63 a–e	27.73 d–l	6.42 bcd	++
I-24	Greyish white	Flat	Smooth	10.21 b–f	23.08 pq	6.08 bcde	+++
I-25	Greyish white	Raised fluffy	Smooth	8.95 f–j	26.053 i–o	6.46 bcd	+++
I-26	Greyish white	Fluffy	Irregular	9.66 c–h	26.15 i–o	6.19 bcde	+++
I-27	Grey	Flat	Smooth	10.10 b–f	30.27 b–e	6.53 bcd	++
I-28	Grey	Flat	Smooth	10.42 b–f	33.51 a	8.46 a	++
I-29	Grey	Flat	Irregular	8.09 h–l	24.99 l–p	6.25 bcd	++
I-30	Grey	Raised fluffy	Irregular	6.52 lm	27.62 e–l	6.293 bcd	+
I-31	Whitish grey	Raised fluffy	Smooth	10.64 a–e	26.59 g–n	6.19 bcde	+++
I-32	Grey	Raised fluffy	Smooth	11.07 abc	30.53 bcd	6.52 bcd	+
I-33	Grey	Flat	Smooth	9.35 d–h	30.76 abc	6.63 bcd	+
I-34	Grey	Flat	Irregular	10.52 a–f	27.37 f–m	6.28 bcd	++
I-35	Greyish white	Raised fluffy	Smooth	10.63 a–e	23.49 opq	6.61 bcd	+++
I-36	Grey	Raised fluffy	Smooth	10.35 b–f	33.08 ab	7.84 a	+
I-37	Dark grey	Flat	Smooth	9.52 c–h	27.77 d–l	6.49 bcd	++
I-38	Grayish white	Raised fluffy	Smooth	10.63 a–e	20.86 q	6.26 bcd	++
I-39	Greyish white	Raised fluffy	Smooth	10.43 b–f	27.75 d–l	5.83 ed	++
I-40	Grey	Raised fluffy	Smooth	10.63 a–e	28.48 c–i	6.89 b	+
I-41	Greyish white	Raised fluffy	Smooth	12.14 a	29.03 c–h	5.96 cde	++
I-42	Greyish white	Flat	Irregular	9.45 c–h	29.53 cdef	6.394 bcd	+++
Mean	-	-	-	9.70 ± 0.22	26.99 ± 0.49	6.41 ± 0.15	-

* Mean of fifteen conidia; Means supported by same letters in a column don’t vary significantly at the Tukey’s Studentized Range’ test at *p* ≤ 0.05. + Low = 2.08 to 16.67 × 105; ++ Medium = 16.67 to 29.17 × 105; +++ High = 29.17 to 41.67 × 105.

**Table 4:** Disease reactions and virulence categories of *C. falcatum* isolates from Bangladesh on infected sugarcane variety, Isd 28.

Sl No.	Isolates	Disease Reactions & Score	Virulence Categories	Geographic Regions
1	I -1	HS (9.0)	Virulent	Rajshahi
2	I-2	HS (8.6)	Virulent	
3	I-3	MS (5.9)	Moderately Virulent	
4	I-6	MR (3.3)	Less Virulent	
5	I-7	MR (3.3)	Less Virulent	
6	I-12	MR (2.7)	Less Virulent	
7	I-16	R (2.0)	Less Virulent	
8	I-18	HS (8.7)	Virulent	
9	I-23	HS (9.0)	Virulent	
10	I-26	HS (8.6)	Virulent	
11	I-27	MR (3.6)	Less Virulent	
12	I-29	MS (5.3)	Moderately Virulent	
13	I-30	MS (5.1)	Moderately Virulent	
14	I-31	HS (9.0)	Virulent	
15	I-32	MR (3.2)	Less Virulent	
16	I-36	MR (2.4)	Less Virulent	
17	I-39	S (6.3)	Virulent	
18	I-5	MR (2.8)	Less Virulent	Rangpur
19	I-19	HS (8.3)	Virulent	
20	I-20	HS (8.5)	Virulent	
21	I-24	MR (2.9)	Less Virulent	
22	I-8	R (1.4)	Less Virulent	Khulna
23	I-11	MR (3.7)	Less Virulent	
24	I-13	MR (2.6)	Less Virulent	
25	I-14	MR (3.1)	Less Virulent	
26	I-15	MS (5.6)	Moderately Virulent	
27	I-17	R (1.8)	Less Virulent	
28	I-21	S (6.6)	Virulent	
29	I-22	MS (4.7)	Moderately Virulent	
30	I-25	MR (3.6)	Less Virulent	
31	I-33	MR (2.8)	Less Virulent	
32	I-40	MR (2.2)	Less Virulent	
33	I-9	MS (4.1)	Moderately Virulent	Dhaka
34	I-10	MR (2.7)	Less Virulent	
35	I-28	MS (5.8)	Moderately Virulent	
36	I-34	MR (2.6)	Less Virulent	
37	I-35	MR (3.5)	Less Virulent	
38	I-37	R (0.8)	Less Virulent	
39	I-38	MR (2.1)	Less Virulent	
40	I-41	R (1.7)	Less Virulent	
41	I-42	R (0.9)	Less Virulent	

**Table 5:** Polymorphism of *C. falcatum* isolates from different locations in Bangladesh generated by 10 ISSR markers.

SL No.	Marker	NAB	NMB	NPB	PPB (%)
1	UBC810	37	2	35	94.59
2	UBC 850	36	0	36	100.00
3	UBC 828	42	1	41	97.62
4	ISSR 7	45	0	45	100.00
5	UBC 860	42	0	42	100.00
6	ISSR 10	43	0	43	100.00
7	UBC 857	31	0	31	100.00
8	UBC 825	42	0	42	100.00
9	UBC-873	46	1	45	97.83
10	ISSR 845	40	0	40	100.00
Mean		40.40	0.40	40.00	99.01

NAB: Number of Amplified Bands; NMB: Number of Monomorphic Bands; NPB: Number of Polymorphic Bands.

**Table 6:** Genetic structure of *C. falcatum* isolates collected from four geographic regions of Bangladesh analyzed using10 ISSR markers.

Populations	PPB (%)	Na	Ne	H	I
Raj	68.81	1.6881	1.2388	0.1610	0.2640
Ran	34.16	1.3416	1.2356	0.1377	0.2021
Khu	55.45	1.5545	1.2324	0.1520	0.2422
Dha	55.20	1.5520	1.2413	0.1577	0.2500
Mean Value	53.40	1.5341	1.2370	0.1521	0.2396
Mean at Species level	99.01	1.9901	1.2425	0.1732	0.2386

Raj: Rajshahi, Ran: Rangpur, Khu: Khulna, Dha: Dhaka, PPB: Percent of polymorphic band Na: Observed, Number of Alleles; Ne: Effective Number of Alleles; H: Nei’ Gene Diversity; I: Shannon’s Index of Diversity.

**Table 7:** Analysis of molecular variance (AMOVA) among and within populations of *C. falcatum* from four regions of Bangladesh.

Source of Variation	Degree of Freedom	Sum of Square	Mean Square	Estimated Variance	Total Variance (%)	*p* Value
Among the populations	3	326.373	108.791	8.260	22%	<0.001
Within the Populations	37	1108.164	29.950	29.950	78%	<0.001
Total	40	1434.537	-	38.211	100%	-

**Table 8:** Pair wise comparisons of genetic identity and genetic distance among the four *C. falcatum* populations based on ISSR markers.

Populations	Raj	Ran	Khu	Dha
**Raj**	****	0.976	0.980	0.991
**Ran**	0.009	****	0.971	0.977
**Khu**	0.023	0.020	****	0.970
**Dha**	0.031	0.030	0.024	****

Raj: Rajshahi, Ran: Rangpur, Khu: Khulna, Dha: Dhaka; Nei unbiased genetic identity (above diagonal) and genetic distance (below diagonal).

## Data Availability

The data presented in this study are available on request from the corresponding author.

## Supplementary Materials

The following are available online at https://www.mdpi.com/article/10.3390/biology10090862/s1, Supplementary Figure S1. Sites sampled for sugarcane red rot disease in four regions of Rangpur, Rajshahi, Dhaka and Khulna in Bangladesh, Supplementary Table S1. Characteristics for the assessment of the disease index for the virulence pattern of C. falcatum against sugarcane. Supplementary Table S2. Colletotrichum falcatum isolates used for phylogenetic analysis and their accession numbers, Supplementary Figure S2. Phylogenetic tree of C. falcatum isolates using Maximum likelihood method based on (a) ITS and (b) β-tubulin sequences. The tree was rooted with Monilochaetes infuscan.ML bootstrap value is indicated at each node, Supplementary Figure S3. Phylogenetic tree of C. falcatum isolates using Maximum Likelihood method based on (a) Actin and (b) GAPDH sequences. The tree was rooted with Monilochaetes infuscan.ML bootstrap value is indicated at each node.

## References

[1] Patel P., Rajkumar B.K., Parmar P., Shah R., Krishnamurthy R. (2018). Assessment of genetic diversity in *Colletotrichum falcatum* Went accessions based on RAPD and ISSR markers. J. Genet. Eng. Biotechnol..

[2] Food and Agricultural Organization of United Nations, Economic and Social Department (2018). The Statistical Division.

[3] Uddin M.S., Talukdar M.I., Rahman M.S. (2013). Screening of red rot genotypes of sugarcane in Bangladesh. Bangladesh J. Sugarcane.

[4] Rafay S.A., Singh V.B. (1957). A new strain of *Glomerella tucumanesis*. Curr. Sci..

[5] Bailey J.A., Jeger M.J. (1992). Colletotrichum: Biology, Pathology and Control.

[6] Talukder M.I., Kamal M.M., Iqbal M., Rahman S. (2010). Bangladesh Akher Rog BalaiSomuho o Tar Pratikar.

[7] Ghazanfar M.U., Kamran S. (2016). Evaluation of Different Plant Extracts against *Colletotrichum falcatum* Causing Red Rot in Sugarcane under Lab Conditions. J. Environ. Agric..

[8] Sengar A.S., Thind K.S., Kumar B., Pallavi M., Gosal S.S. (2009). In vitro selection at cellular level for red rot resistance in sugarcane (*Saccharum* sp.). Plant Growth Regul..

[9] Kumar N., Jhang T., Satyavir N., Sharma T.R. (2011). Molecular and Pathological Characterization of *Colletotrichum falcatum* Infecting Subtropical Indian Sugarcane. J. Phytopathol..

[10] Duttamajumdar S.K. (2008). Red Rot of Sugarcane.

[11] Freeman S., Shabi E., Katan T. (2000). Characterization of *Colletotrichum acutatum* causing anthracnose of anemone (*Anemone coronaria* L.). Appl. Environ. Microbiol..

[12] Hyde K.D., Cai L., McKenzie E.H.C., Yang Y.L., Zhang J.Z., Prihastuti H. (2009). *Colletotrichum*: A catalogue of confusion. Fungal Divers..

[13] Bharti Y.P., Vishwakarma S.K., Kumar A., Singh A., Sharma M.L., Shukla D.N. (2012). Physiological and Pathological Aspects of some new isolates of *Colletotricum falcatum* causing Red rot disease in *Saccharum* spp. complex. Acta Phytopathol. Entomol. Hung..

[14] Cai L., Hyde K.D., Taylor P.W.J., Weir B., Waller J., Abang M.M., Prihastuti H. (2009). A polyphasic approach for studying *Colletotrichum*. Fungal Divers..

[15] Martin K.J., Rygiewicz P.T. (2005). Fungal-specific PCR primers developed for analysis of the ITS region of environmental DNA extracts. BMC Microbiol..

[16] Talhinhas P., Sreenivasaprasad S., Neves-Martins J., Oliveira H. (2005). Molecular and phenotypic analyses reveal association of diverse *Colletotrichum acutatum* groups and a low level of *C. gloeosporioides* with olive anthracnose. Appl. Environ. Microbiol..

[17] McDonald B.A., Linde C. (2002). The population genetics of plant pathogens and breeding strategies for durable resistance. Euphytica.

[18] Rangaswami G. (1958). An agar blocks technique for isolating soil microorganisms with special reference to Pythiaceous fungi. Sci. Cult..

[19] Pandey V., Shukla D.N. (2017). Morphological Studies on Red Rot of Sugarcane from Hardoi District of Uttar Pradesh. Int. J. Agric. Innov. Res..

[20] Abbas H., Anwar S.A., Javed N., Iqbal M.A., Abid N. (2010). Morphological variability among isolates of *Colletotrichum falcatum* Went; infecting four cultivars of sugarcane. Pak. J. Phytopathol..

[21] Sangdit P., Leksomboon C., Lertsrutaiyotin R. (2014). Cultural, morphological and pathological characterization of *Colletotrichum falcatum* causing red rot disease of sugarcane in Thailand. Agric. Nat. Resour..

[22] Prema R.T., Raguchander T., Kalaimani T. (2013). Morphological characterization and reaction of partial purified toxin of sugarcane red rot pathogen *Colletotrichum falcatum* collected from Southern India. Int. J. Agric. Sci..

[23] Kaur R., Kumar B., Vikal Y., Sanghera G.S. (2014). Genetic Diversity among *Colletotrichum falcatum* Isolates Causing Red Rot of Sugarcane in Subtropical Region of India. Not. Sci. Biol..

[24] Rahman M.S., Talukder M.I., Iqbal M., Begum F., Khatun S. (2009). Reactions of some sugarcane genotypes against red rot (*Colletotrichum falcatum* Went.) disease. Indian Sugar.

[25] Srinivasan K.V., Bhat N.R. (1961). Red rot of sugarcane criteria for grading resistance. J. Indian Bot. Soc..

[26] Rahman S., Alam S.M., Joarder O.I., Alam S. (1998). Red rot incidence of sugarcane in Bangladesh. Bangladesh J. Sugarcane.

[27] Shukla R.K., Tripathi D.N.P., Pundey A.K., Singh P.O., Singh S.B. (2005). A new mid late maturing and red rot resistant cultivar for high tonnage and recovery. Indian Sugar.

[28] Viswanathan R. (2017). Pathogen virulence in sugarcane red rot pathogen versus varieties in cultivation: Classical case of loss in virulence in the pathotype CF06 (Cf671). Sugar Tech..

[29] Viswanathan R., Padmanaban P., Selvakumar R. (2019). Emergence of new pathogenic variants in *Colletotrichum falcatum,* stalk infecting ascomycete in sugarcane: Role of host varieties. Sugar Tech..

[30] Lin Y.H., Chang J.Y., Liu E.T., Chao C.P., Huang J.W., Chang P.F.L. (2009). Development of a molecular marker for specific detection of *Fusarium oxysporum* f. sp.. Cubenserace 4 Eur. J. Plant Pathol..

[31] White T.J., Bruns T., Lee S., Taylor J.W., Innis M.A., Gelfand D.H., Sninsky J.J., White T.J. (1990). Amplification and direct sequencing of fungal ribosomal RNA genes for phylogenetics. PCR Protocols: A Guide to Methods and Applications.

[32] Gardes M., Bruns T.D. (1993). ITS primers with enhanced specificity for Basidiomycetes application to the identification of mycorrhizae and rusts. Mol. Ecol..

[33] Wang R.Y., Gao B., Li X.H., Ma J., Chen S.L. (2014). First report of *Fusarium solani* causing *Fusarium* root rot and stem canker on storage roots of sweet potato in China. Plant Dis..

[34] Carbone I., Kohn L.M. (1999). A method for designing primer sets for speciation studies in filamentous ascomycetes. Mycologia.

[35] Templeton M.D., Rikkerink E.H.A., Solon S.L., Crowhurst R.N. (1992). Cloning and molecular characterization of the glyceraldehyde-3-phosphate dehydrogenaseen coding gene and cDNA from the plant pathogenic fungus *Glomerella cingulata*. Gene.

[36] Hall T.A. (1999). BioEdit: A user-friendly biological sequence alignment editor and analysis program for Windows 95/98/NT. Nucleic Acids Symp. Ser..

[37] Hall T., Biosciences I., Carlsbad C. (2011). BioEdit: An important software for molecular biology. GERF Bull. Biosci..

[38] Kumar S., Stecher G., Tamura K. (2016). MEGA7: Molecular Evolutionary Genetics Analysis version 7.0 for bigger datasets. Mol. Biol. Evol..

[39] Kimura M. (1980). A simple method for estimating evolutionary rate of base substitutions through comparative studies of nucleotide sequences. J. Mol. Evol..

[40] Gupta M., Chyi Y.S., Severson R.J., Owen J.L. (1994). Amplification of DNA markers from evolutionary diverse genomes using single primers of simple sequence repeats. Theory Appl. Genet..

[41] Arade P.C., Singh P., Mahatma M. (2014). Characterization of *Colletotrichum falcatum* Went. Causing Red Rot in Sugarcane *Saccharum* complex. Bioscan.

[42] Kaewchai S., Wang H.K., Lin F.C., Hyde K.D., Soytong K. (2009). Genetic variation among isolates of *Rigidoporus*
*microporus* causing white root disease of rubber trees in southern Thailand revealed by ISSR markers and pathogenicity. Afr. J. Microbiol. Res..

[43] Soytong K., Kaewchai S. (2014). Biological control of white root of rubber trees using *Chaetomium cupreum*. J. Agric. Technol..

[44] Rohlf F.J. (1998). NTSYSpc Numerical Taxonomy and Multivariate Analysis System Version 2.0 User Guide.

[45] Peakall R., Smouse P.E. (2012). GenAlEx 6.5: Genetic Analysis in Excel. Population genetic software for teaching and research an update. Bioinformatics.

[46] Nei M., Li W.H. (1979). Mathematical model for studying genetic variation in terms of restriction endonucleases. Proc. Natl. Acad. Sci. USA.

[47] Kimura M., Crow J.F. (1964). The number of alleles that can be maintained in a finite population. Genetics.

[48] Yeh F.C., Boyle T.J.B. (1997). Sample genetic analysis of co-dominant and dominant markers and quantitative traits. Belg. J. Bot..

[49] McDermott J.M., McDonald B.A. (1993). Gene flow in plant pathosystems. Annu. Rev. Phytopathol..

[50] Wright S. (1951). The genetical structure of populations. Ann. Eugen..

[51] Malathi P., Viswanathan R., Ramesh Sundar A., Padmanaban P., Prakasam N., Mohanraj D., Jothi R. (2011). Phylogenetic analysis of *Colletotrichum falcatum* isolates causing red rot in sugarcane. J. Sugarcane Res..

[52] Rahman M.S., Khatun K., Rahman K. (2016). Sugarcane and sugar industry in Bangladesh: An Overview. Sugar Tech..

[53] Viswanathan R., Padmanaban P., Mohanraj D., Jothi R. (2000). Indirect-ELISA technique for the detection of the red rot pathogen in sugarcane (*Saccharum* spp. hybrid) and resistance screening. Indian J. Agric. Sci..

[54] Mishra M.K., Behera B. (2009). Pathogenic and molecular Variability of *Colletotrichum falcatum* Went. Isolates from sugarcane with red rot disease symptoms. J. Crop. Sci. Biotechnol..

[55] Prittesh P., Amaresan N., Rushabh S., Krishnamurthy R., Bhasker V.V. (2016). Isolation and pathogenic variability of *Colletotrichum falcatum* causing red rot in sugarcane. J. Plant Dis. Prot..

[56] Malathi P., Viswanathan R. (2012). Variations in *Colletotrichum falcatum* red rot pathogen of sugarcane in relation to host resistance. Sugar Tech..

[57] Wang L., Liu L.M., Wang Z.G., Huang S.W. (2013). Genetic Structure and Aggressiveness of *Rhizoctonia*
*solani* AG 1-IA, the Cause of Sheath Blight of Rice in Southern China. J. Phytopathol..

[58] Mahmodi F., Kadir J., Puteh A. (2013). Genetic Diversity and Pathogenic Variability of *Colletotrichum*
*truncatum* Causing Anthracnose of Pepper in Malaysia. J. Phytopathol..

[59] Aktaruzzaman M., Afroz T., Lee Y.G., Kim B.S. (2018). Post-harvest anthracnose of papaya caused by *Colletotrichum truncatum* in Korea. Eur. J. Plant Pathol..

[60] Jayawardena R.S., Hyde K.D., Damm U., Cai L., Liu M., Li X.H., Yan J.Y. (2016). Notes on currently accepted species of *Colletotrichum*. Mycosphere.

[61] Hyde K.D., Cai L., Cannon P.F., Crouch J.A., Crous P.W., Damm U., Goodwin P.H. (2009). *Colletotrichum* names in current use. Fungal Divers..

[62] Cannon P.F., Damm U., Johnston P.R., Weir B.S. (2012). *Colletotrichum* current status and future directions. Stud. Mycol..

[63] Hossain M.I., Ahmad K., Siddiqui Y., Saad N., Rahman Z., Haruna A.O., Bejo S.K. (2020). Current and Prospective Strategies on Detecting and Managing *Colletotrichum falcatum* Causing Red Rot of Sugarcane. Agronomy.

[64] Shahnazi S., Meon S., Ebrahimi M. (2013). Characterisation, dierentiation and biochemical diversity of *Fusarium solani* and *Fusarium proliferatum* based on cellular fatty acid profiles. Arch. Phytopathol. Plant Prot..

[65] Oghenekaro A.O., Miettinen O., Omorusi V.I., Evueh G.A., Farid M.A., Gazis R., Asiegbu F.O. (2014). Molecular phylogeny of *Rigidoporus microporus* isolates associated with white rot disease of rubber trees (*Hevea brasiliensis*). Fungal Biol..

[66] Hyde K.D., Nilsson R.H., Alias S.A., Ariyawansa H.A., Blair J.E., Cai L., Gorczak M. (2014). One stop shop: Backbones trees for important phytopathogenic genera: I. Fungal Divers..

[67] Gruning C.R., Sieber T.N., Rogers S.O., Holdenrieder O. (2001). Characterization of dark septate endophytic fungi (DSE) using inter simple sequence repeat anchored polymerase chain reaction (ISSR PCR) amplification. Mycol. Res..

[68] Muller M.M., Valjakka R., Suokka A., Hantula J. (2001). Diversity of endophytic fungi of single Norway spruce needles and their role as pioneer decomposers. Mol. Ecol..

[69] Rodrigues K.F., Sieber T.N., Grünig C.R., Holdenrieder O. (2004). Characterization of *Guignardia mangiferae* isolated from tropical plants based on morphology, ISSR-PCR amplifications and ITS1-5.8S-ITS2 sequences. Mycol. Res..

[70] Rampersad S.N. (2013). Genetic structure of *Colletotrichum*
*gloeosporioides* sensual to isolates infecting papaya inferred by multilocus ISSR markers. Phytopathology.

[71] Zhou S., Smith D.R., Stanosz G.R. (2001). Differentiation of *Botryosphaeria* species and related anamorphic fungi using inter simple or short sequence repeat (ISSR) fingerprinting. Mycol. Res..

[72] McDonald B.A. (1997). The population genetics of fungi: Tools and techniques. Phytopathology.

[73] Bennett R.S., Milgroom M.G., Bergstrom G.C. (2005). Population structure of seed borne *Phaeosphaerian odorum* on New York wheat. Phytopathology.

[74] Weeds P.L., Chakraborty S., Fernandes C.D., d’ACharchar M.J., Ramesh C.R., Kexian Y., Kelemu S. (2003). Genetic diversity in *Colletotrichum gloeosporioides* from *Stylosanthes* spp. at centers of origin and utilization. Phytopathology.

[75] Alsultan W., Vadamalai G., Khairulmazmi A., Saud H.M., Al-Sadi A.M., Rashed O., Jaaffar A.K.M., Nasehi A. (2019). Isolation, identification and characterization of endophytic bacteria antagonistic to *Phytophthora palmivora* causing black pod of cocoa in Malaysia. Eur. J. Plant Pathol..

[76] Taheri P., Gnanamanickam S., Höfte M. (2007). Characterization, genetic structure, and pathogenicity of *Rhizoctonia* spp. associated with rice sheath diseases in India. Phytopathology.

[77] McDonald B.A. (2004). Population genetics of plant pathogens. Plant Health Instr..

[78] Milgroom M.G., Peever P.T. (2003). Population biology of plant pathogens: The synthesis of plant disease epidemiology and population genetics. Plant Dis..

[79] Rashed O., Abdullah N.A., Alsultan W., Misawa T., Ahmad K., Kutawa A.B. (2021). Characterization of inter and intra anastomosis group of *Rhizoctonia* spp. isolated from different crops in Peninsular Malaysia. Trop. Plant Pathol..

