# Supplementary material for: Phylogenetic Analysis and Genetic Diversity of Colletotrichum falcatum Isolates Causing Sugarcane Red Rot Disease in Bangladesh
Source: Biology (Basel). 2021 Sep 3;10(9):862. doi: 10.3390/biology10090862 (PMC8467384; doi:10.3390/biology10090862)
Supplement: Supplementary file 1 [file biology-10-00862-s001.zip › biology-1274179-supplementary.pdf]

Supplementary materials (Table and Figures)

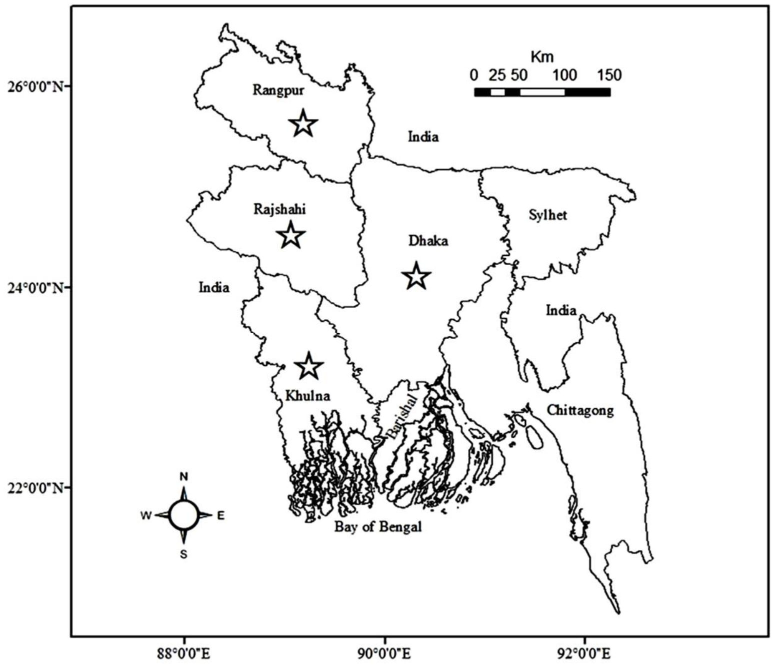

Figure. S1. Sites sampled for sugarcane red rot disease in four regions of Rangpur, Rajshahi, Dhaka and Khulna in Bangladesh . Note:☆indicated the sampling sites

Table S1. Characteristics for the assessment of the disease index for the virulence pattern of *C. falcatum* against sugarcane

Sl. No.	Characters	Numerical rating
1.	**Width of lesions above the inoculated internode (0-3)**	
	i) No lesion or very rare lesion developed	0
	ii) Rare lesion (one third or less spread of full width)	1
	iii) Medium lesion (one third to two third spread of full width)	2
	iv) High lesion (Covered the full width)	3
2.	**Nodal Transgression (0-3)**	
	i) Lesion restricted in the inoculated internodes'	0
	ii) Crossed one node	1
	iii) Crossed two nodes	2
	iv) Crossed three or more nodes	3
3.	**Presence or absence of white patches (0-2)**	
	i) White patches absent	0
	ii) White patch restricted	1
	iii) White patch progressive	2
4.	**Top Conditions (0-1)**	
	i) Green	0
	ii) Yellow/ Dry	1

Table S2. *Colletotrichum falcatum* isolates used for phylogenetic analysis and their accession numbers.

Species	Isolates/Strain	Country	Gene Bank Accession numbers			
			ITS	β-tubulin	ACT	GADHP
C. falcatum	I-1	Bangladesh	MN636336	MN643093	MN643114	MN643135
C. falcatum	I-2	Bangladesh	MK850182	MK867399	MK867379	MK867419
C. falcatum	I-3	Bangladesh	MN636355	MN643094	MN643115	MN643136
C. falcatum	I-5	Bangladesh	MK850183	MK867400	MK867380	MK867420
C. falcatum	I-6	Bangladesh	MN636337	MN643095	MN643116	MN643137
C. falcatum	I-7	Bangladesh	MK850174	MK867401	MK867381	MK867421
C. falcatum	I-8	Bangladesh	MK850192	MK867402	MK867382	MK867422
C. falcatum	I-9	Bangladesh	MK850179	MK867403	MK867383	MK867423
C. falcatum	I-10	Bangladesh	MK850175	MK867404	MK867384	MK867424
C. falcatum	I-11	Bangladesh	MK850191	MK867405	MK867385	MK867425
C. falcatum	I-12	Bangladesh	MN636338	MN643096	MN643117	MN643138
C.falcatum	I-13	Bangladesh	MK850187	MK867406	MK867386	MK867426
C. falcatum	I-14	Bangladesh	MN636339	MN643097	MN643118	MN643139
C. falcatum	I-15	Bangladesh	MK850176	MK867407	MK867387	MK867427
C. falcatum	I-16	Bangladesh	MN636340	MN643098	MN643119	MN643140
C. falcatum	I-17	Bangladesh	MK850184	MK867408	MK867388	MK867428
C. falcatum	I-18	Bangladesh	MK850185	MK867409	MK867389	MK867429
C. falcatum	I-19	Bangladesh	MK850180	MK867410	MK867390	MK867430
C. falcatum	I-20	Bangladesh	MN636341	MN643099	MN643120	MN643141
C. falcatum	I-21	Bangladesh	MN636342	MN6430100	MN643121	MN643142
C. falcatum	I-22	Bangladesh	MN636343	MN6430101	MN643122	MN643143
C. falcatum	I-23	Bangladesh	MK850188	MK867411	MK867391	MK867431
C.falcatum	I-24	Bangladesh	MK850181	MK867412	MK867392	MK867432
C. falcatum	I-25	Bangladesh	MN636344	MN6430102	MN643123	MN643144
C. falcatum	I-26	Bangladesh	MN636345	MN6430103	MN643124	MN643145
C. falcatum	I-27	Bangladesh	MK850177	MK867413	MK867393	MK867433
C. falcatum	I-28	Bangladesh	MK850189	MK867414	MK867394	MK867434
C. falcatum	I-29	Bangladesh	MN636346	MN6430104	MN643125	MN643146
C. falcatum	I-30	Bangladesh	MN636347	MN6430105	MN643126	MN643147
C. falcatum	I-31	Bangladesh	MN636348	MN6430106	MN643127	MN643148
C. falcatum	I-32	Bangladesh	MN636349	MN6430107	MN643128	MN643149
C. falcatum	I-33	Bangladesh	MN636350	MN6430108	MN643129	MN643150
C. falcatum	I-34	Bangladesh	MK850178	MK867415	MK867395	MK867435
C. falcatum	I-35	Bangladesh	MK850190	MK867416	MK867396	MK867436
C. falcatum	I-36	Bangladesh	MN636351	MN6430109	MN643130	MN643151
C. falcatum	I-37	Bangladesh	MN636352	MN6430110	MN643131	MN643152
C. falcatum	I-38	Bangladesh	MK850186	MK867417	MK867397	MK867437
C. falcatum	I-39	Bangladesh	MN636356	MN6430111	MN643132	MN643153
C. falcatum	I-40	Bangladesh	MK850193	MK867418	MK867398	MK867438
C. falcatum	I-41	Bangladesh	MN636353	MN6430112	MN643133	MN643154
C. falcatum	I-42	Bangladesh	MN636354	MN6430113	MN643134	MN643155
C. falcatum	Cf01	India	KU220959	-	-	-
C. falcatum	Cf 02	India	KU220960	-	-	-
C. falcatum	RR01	India	KU220961	-	-	-
C. falcatum	RR 02	India	KU220962	-	-	-
C. falcatum	RR 03	India	KU220963	-	-	-
C. falcatum	1802020746Q	China	MK937676	-	-	-
C. falcatum	LC885	China	HM171677	-	HM171665	HM171671
C. falcatum	LC03	China	KU933924	-	-	-
C. falcatum	MAFF 306170	Japan	AB462376	-	-	-
C. falcatum	CBS 147945	Netherland	JQ005772	-	-	-
C. falcatum	Strain	Mexico	AF487427	-	-	-
C. falcatum	CBS 127945	USA	NR144793			
C. falcatum	BRIP28041	Hong Kong	-	DQ195740	-	-
C. falcatum	Strain	Hong Kong	-	DQ195756	-	-
C. falcatum	CBS:147945	Netherland	-	-	JQ005835	-
C. falcatum	CP-CC018	Mexico	-	-	KP895578	-
C. falcatum	CP-CC019	Mexico	-	-	KP895579	-
C. falcatum	CP-CC020	Mexico	-	-	KP895580	-
C. falcatum	Strain	Thailand	-	-	FJ907431	-
C. falcatum	Cf86032C	India	-	-	FJ008081	-
C. falcatum	Cf90063	India	-	-	FJ008096	-
C. falcatum	Cf86032T	India	-	-	FJ008097	FJ002018
C. falcatum	Cf94101K	India	-	-	FJ008098	FJ002019
C. falcatum	Cf64-8	India	-	-	FJ008099	FJ002020
C. falcatum	Cf64-11	India	-	-	FJ008100	FJ002021
C. falcatum	Cf767A	India	-	-	-	FJ002008
C. falcatum	Cf767B	India	-	-	-	FJ002011
C. falcatum	Cf89V74	India	-	-	-	FJ002000
C. falcatum	Strain	Thailand	-	-	-	FJ972585
C. endophytum	CGMCC 3.15108	China	JX625177	JX62526	KC843533	KC843521
C.graminicola	CBS 130836	Netherlands	JQ005767	JQ005851	JQ005830	-

C.eleusines	MAFF 511155	Netherlands	JX519218	JX519243	JX519234	-
C.gloeosporioides	ICMP: 17821	Italy	JX010152	JX010445	JX009531	JX010056
C.fructicola	ICMP:18613	Israel	JX010167	JX010388	JX009491	JX009998
C.acutatum	CBS:112996	Netherlands	JQ005776	JQ005860	JQ005839	JQ948677
C.austral	CBS:116478	Netherlands	JQ948455	JQ950106	JQ949776	JQ948786

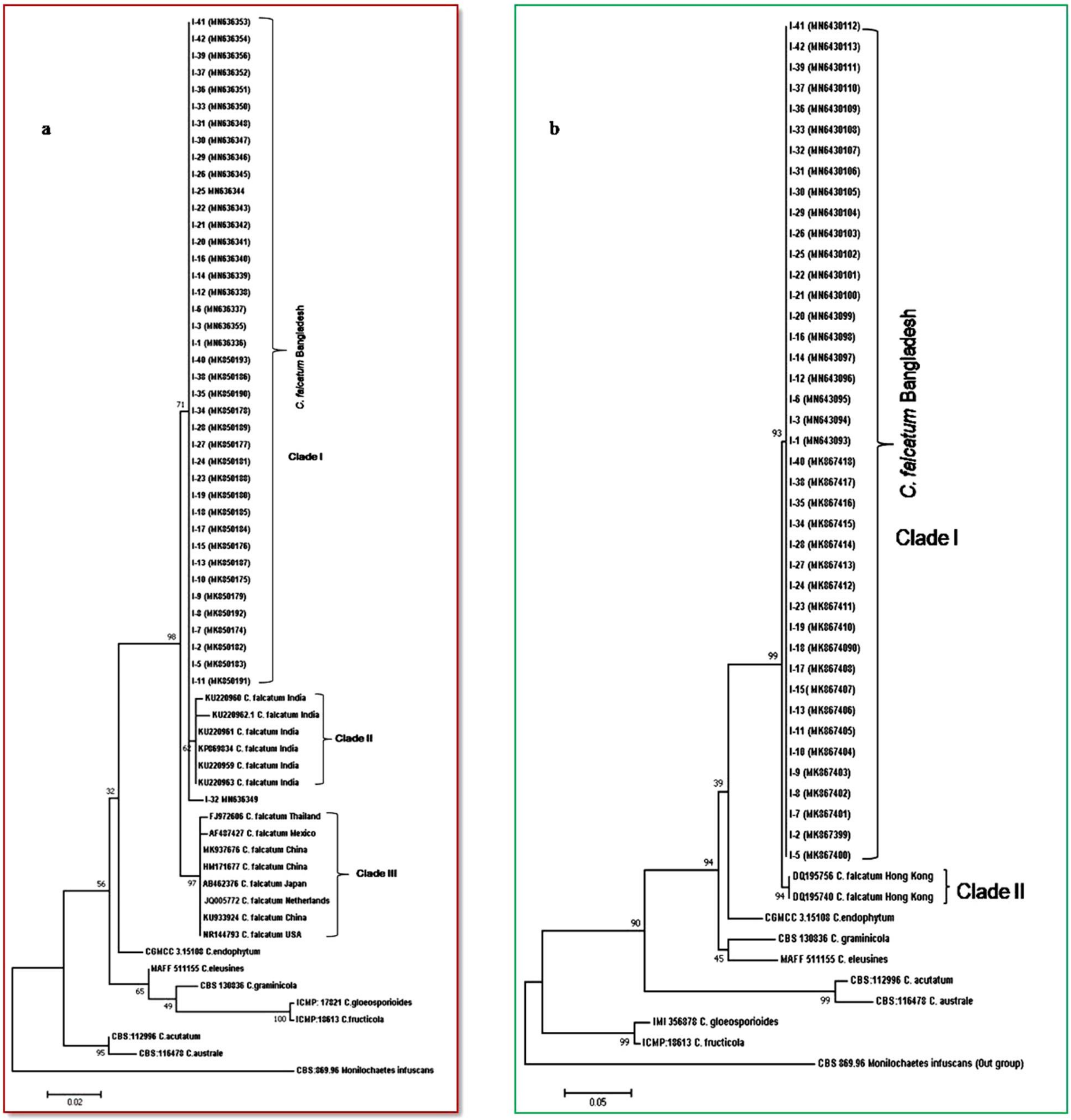

Figure S2. Phylogenetic tree of *C. falcatum* isolates using Maximum Likelihood method based on (a) ITS and (b) β-tubulin sequences. The tree was rooted with *Monilochaetes infuscan*.ML bootstrap value is indicated at each node.

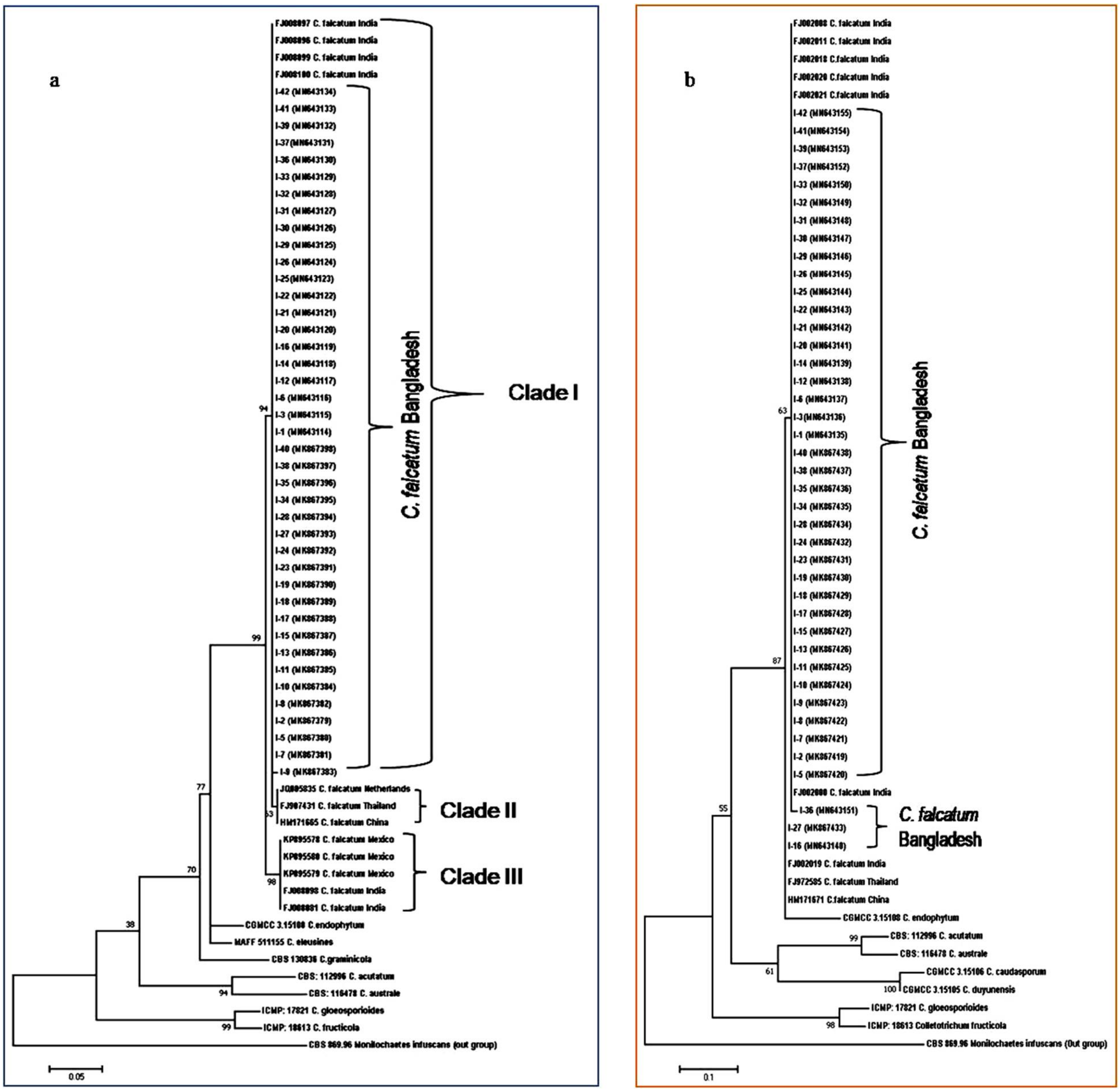

Figure S3. Phylogenetic tree of *C. falcatum* isolates using Maximum Likelihood method based on (a) Actin and (b) GAPDH sequences. The tree was rooted with *Monilochaetes infuscan*.ML bootstrap value is indicated at each node.
